# Improving power in functional magnetic resonance imaging by moving beyond cluster-level inference

**DOI:** 10.1073/pnas.2203020119

**Published:** 2022-08-04

**Authors:** Stephanie Noble, Amanda F. Mejia, Andrew Zalesky, Dustin Scheinost

**Affiliations:** ^a^Department of Radiology and Biomedical Imaging, Yale School of Medicine, New Haven, CT 06519;; ^b^Department of Statistics, Indiana University Bloomington, Bloomington, IN 47408;; ^c^Melbourne Neuropsychiatry Centre, The University of Melbourne, Melbourne, VIC 3010, Australia;; ^d^Department of Biomedical Engineering, The University of Melbourne, Melbourne, VIC 3010, Australia;; ^e^Department of Biomedical Engineering, Yale School of Medicine, New Haven, CT 06520;; ^f^Interdepartmental Neuroscience Program, Yale University, New Haven, CT 06520;; ^g^Department of Statistics and Data Science, Yale University, New Haven, CT 06511;; ^h^Child Study Center, Yale School of Medicine, New Haven, CT 06519

**Keywords:** fMRI, power, inference, network, empirical

## Abstract

Inference in neuroimaging typically occurs at the level of focal brain areas or circuits. Yet, increasingly, well-powered studies paint a much richer picture of broad-scale effects distributed throughout the brain, suggesting that many focal reports may only reflect the tip of the iceberg of underlying effects. How focal versus broad-scale perspectives influence the inferences we make has not yet been comprehensively evaluated using real data. Here, we compare sensitivity and specificity across procedures representing multiple levels of inference using an empirical benchmarking procedure that resamples task-based connectomes from the Human Connectome Project dataset (∼1,000 subjects, 7 tasks, 3 resampling group sizes, 7 inferential procedures). Only broad-scale (network and whole brain) procedures obtained the traditional 80% statistical power level to detect an average effect, reflecting >20% more statistical power than focal (edge and cluster) procedures. Power also increased substantially for false discovery rate– compared with familywise error rate–controlling procedures. The downsides are fairly limited; the loss in specificity for broad-scale and FDR procedures was relatively modest compared to the gains in power. Furthermore, the broad-scale methods we introduce are simple, fast, and easy to use, providing a straightforward starting point for researchers. This also points to the promise of more sophisticated broad-scale methods for not only functional connectivity but also related fields, including task-based activation. Altogether, this work demonstrates that shifting the scale of inference and choosing FDR control are both immediately attainable and can help remedy the issues with statistical power plaguing typical studies in the field.

## Significance

Localizing cognitive function to distinct brain areas has been a mainstay of human brain research since early reports that focal injuries produce changes in behavior. Yet, accumulating evidence shows that areas do not act in isolation. Here, we evaluate the practical implications of the localizationist perspective by comparing the performance of localizing versus broad-scale statistical procedures in real connectome data (1,000 subjects performing 7 tasks). We find that popular localizing procedures miss substantially more true effects than simple broad-scale procedures. By highlighting the power of simple alternatives, we argue that moving beyond localization is viable and can help unlock opportunities for human neuroscience discovery.

Functional magnetic resonance imaging (fMRI) is a cornerstone technique for exploring the living human brain. Most fMRI research is geared toward pinpointing specific brain areas or circuits (i.e., small “clusters” of neighboring voxels or edges) (1, 2)* associated with behaviors, traits, and other phenotypic information. While performing inference at the cluster level accurately reflects some properties of the underlying signal, even decades ago the designers of this approach remarked that more distributed models may better capture the underlying biology (1). Recent work examining datasets that are larger—and deeper—than ever has begun to reveal that task-related activity involves processes occurring in concert throughout the brain (3–5). Large groups of edges across the brain have also been demonstrated to act in concert (6, 7). Yet, despite the emergence of broader-scale methods for inference (8–11) and prediction (12, 13), cluster-level inference remains the primary workhorse for typical fMRI studies.

Given the distributed nature of neural processes, moving to a broader level of inference that spans widespread brain areas may increase our ability to detect effects (i.e., “power” or “sensitivity”). This is critical since the field has recently acknowledged an endemic lack of statistical power in typical fMRI studies (4, 14, 15). Underpowered studies not only are ill-equipped to detect effects that do exist but also lead to findings that do not replicate or only uncover a small tip of the iceberg of true effects. Many have rightly advocated for a variety of approaches to improve power and reproducibility (e.g., increasing sample sizes, designing tasks that elicit more robust responses). However, relatively little attention has been paid to how simply redefining the level of inference may improve power, a step that can be readily adopted by typical researchers.

Using functional connectivity data, we comprehensively explored how power changes with the spatial scale of inference at the level of edges, clusters, large-scale networks, and the whole brain. We simultaneously evaluated key measures of specificity reflecting the ability to filter out “false positives” and hone in on real effects and how results changed when varying the error measure targeted for multiple comparison correction. Critically, designating a full dataset as the population of interest enabled us to fully determine “ground truth” effects while preserving the structure of real data.

Altogether, broader levels of inference provided substantially greater power to detect known empirical effects. In fact, at our smallest sample size (*n* = 40, almost double the typical (15, 16) sample size of *n* = 25), the power to detect an average effect at the edge and cluster levels was nearly half that of the network and whole-brain levels. Even for the largest sample size measured here (*n* = 120), cluster-level inference still missed effects in more than half of the connectome. We found that the gain in power for broader-level inference is closely related to the widespread nature of ground truth effects in a task-based paradigm and that this comes at a relatively modest loss in specificity. All approaches further benefited from more permissive targets for multiple testing correction—particularly smaller-scale approaches—and we argue that the loss in specificity remains reasonable for many typical studies relative to the gain in power. We finally discuss how we expect these findings from functional connectivity to generalize to the task-based activation context and beyond, including other fields with similar widespread dependence in their data.

## Results

**Fig. 1.**
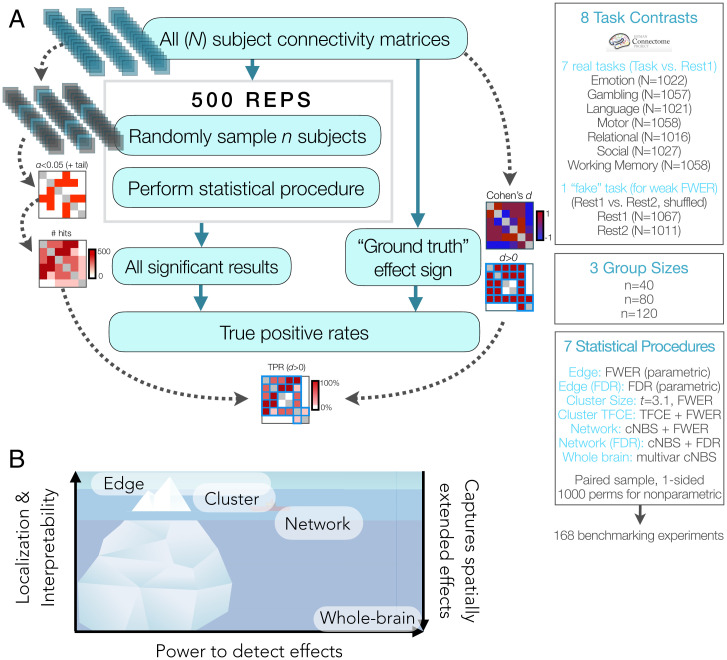
Overview of methods and levels of inference. (*A*) Benchmarking procedure for estimating true positive rates (TPRs) and parameters. (*B*) Tradeoff between the ability to localize and interpret results and the power to detect effects in typical sample sizes based on the present benchmarking study, along with the extent to which each method captures spatially extended effects.

Drawing upon recent computational frameworks for benchmarking fMRI statistical procedures in large, real datasets (3, 17), including our previous work in the task-based activation context (4), we empirically estimated power across levels of inference by resampling functional connectomes derived from the Human Connectome Project (HCP) S1200 dataset (methods summary in Fig. 1; *Methods* for details). Seven procedures were used to perform inference at each level, as follows: “edge,” “edge false discovery rate (FDR),” “cluster size,” “cluster threshold-free cluster enhancement (TFCE),” “network,” “network (FDR),” and “whole brain.” Procedures controlled either the chance of at least one false positive (i.e., familywise error rate [FWER]) or the expected proportion of false positives relative to all positives (i.e., FDR). At each resampling repetition, differences between task and rest connectivity were estimated for a paired sample and compared with the full sample ground truth dataset. “True positives” were defined as detections (i.e., significant edges, clusters, etc.) in the direction matching the ground truth effect sign (akin to refs. 4, 11), and false positives were defined as detections in the opposite direction as the ground truth or during a “fake task contrast” (i.e., REST1 versus REST2, shuffled). Note that the distinction between true and false positives rests on the ground truth effect sign, which is treated as exactly determined here for the purpose of benchmarking. While sign errors may occur in the full dataset particularly for small magnitude effects, the fact that most effects are found to be significant based on the full dataset mitigates this concern (*SI Appendix*, *SI Results 1. Limitations in generalizability of ground truth and performance measures*, for further discussion of this and other issues of generalizability). True and false positives were then used to calculate several measures of performance (*SI Appendix*, Fig. 1). Benchmarking experiments were conducted for each of the seven task scans available in the dataset, and three group sizes were chosen for resampling to span from high typical to moderate sample sizes for this field (*n* = 40, 80, 120) (15, 16).

**Fig. 2.**
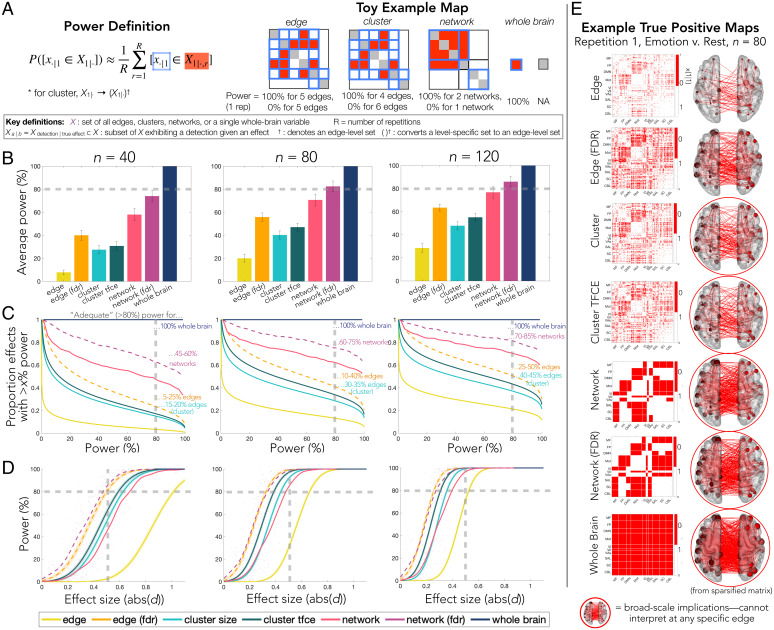
Power across levels of inference. (*A*) Definition of power and toy example. Note that power is calculated at the level of each inferential procedure except cluster-level procedures, for which a true positive is defined at the edge level. Toy examples show results only for a single toy repetition and count only unique edges (e.g., lower triangle only; NA, not applicable). An abridged list of definitions for key terms and notation is provided; for details, see *SI Appendix*, Fig. 1 and *Supplemental Method 4*. (*B*–*D*) Results from the following measures are shown at three sample sizes, averaged across all seven tasks. The commonly targeted β = 80% power threshold is indicated by the dashed gray line. (*B*) Average power to detect an effects (e.g., for edge procedure, mean power across all edges). Bar heights depict the average across all tasks and error bars depict SEM across tasks. (*C*) Proportion of effects exceeding each power level across all tasks. (*D*) Relationship between power and effect size, with a medium effect size of *d* = 0.5 indicated by a dashed gray line (effect size distributions provided in Fig. 3*B*). (*E*) Example true positive maps from the first repetition of the emotion vs. rest contrast at *n* = 80 shown via matrix and glass brain (sparsified for visualization by uniformly selecting 25% nodes). While edges are shown to illustrate the spatial extent of results for interpretation, results that cannot be interpreted at any particular edge but instead at a broader scale have been circled in red. MF, medial frontal; FP, frontoparietal; DMN, default mode; Mot, Motor; VI, visual I; VII, visual II; VAs, visual association; Lim, limbic; BG, basal ganglia; CBL, cerebellum.

Overall, the average power to detect a ground truth effect (Fig. 2*A*) was substantially larger for broader levels of inference and FDR control (Fig. 2*B* and *SI Appendix*, Fig. 2*A*). Only network- and whole-brain-level approaches attained or surpassed “adequate” power, defined here as the commonly targeted β = 80% power level. The whole-brain-level procedure in particular detected all effects even at the smallest sample size. The other approaches ranged from 10% (edge, *n* = 40) to 65% (edge FDR, *n* = 120) average power. The difference between procedures was particularly evident in the smallest group measured (*n* = 40), which is a sample size yet above average for the field. The gap between approaches decreased with larger samples, although a sample larger than the largest measured here (*n* = 120) would be needed to achieve adequate power to detect the average effect for edge- and cluster-level procedures. However, the choice of error rate under control also plays a role; the gain in power for FDR-controlling procedures relative to FWER-controlling procedures was substantial enough that edge-level FDR control resulted in greater power than cluster-level FWER-controlling procedures. A similar pattern of results but lower power overall was observed for weaker and sparser ground truth maps (*SI Appendix*, Fig. 10).

We also examined the proportion of effects that were adequately powered (Fig. 2*C* and *SI Appendix*, Fig. 2*B*). At the more typical sample size (*n* = 40), adequate power was obtained for a quarter or fewer of edges when using edge- and cluster-level procedures. While this represents a substantial number of edges—a quarter of the connectome is nearly 9,000 edges—it also implies that three-quarters or more of the connectome cannot be detected at desirable rates. In contrast, adequate power was obtained for more than half of the network-level effects at *n* = 40, and again, all whole-brain effects were consistently detected. Although the gap between approaches decreased with sample size, the more focal approaches remained inadequately powered for over half the connectome with even the largest sample size.

### How the Spatial Extent of Ground Truth Effects Influences Power.

**Fig. 3.**
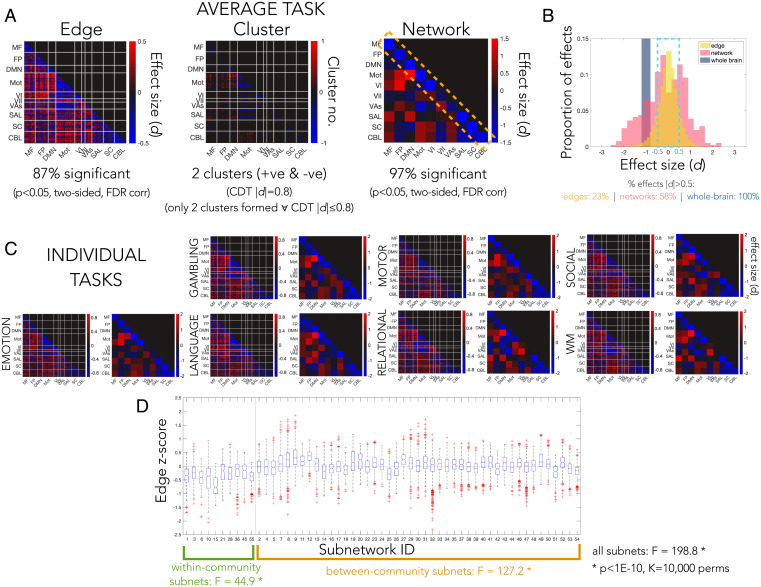
Spatial extent of effects in the full ground truth dataset. (*A*) Edge-, cluster-, and network-level effects. The average effect size and average number of significant effects across the seven tasks (task-rest) are shown for the edge and network level (*P* < 0.05, two-sided *t* test, FDR corrected via Storey). For the cluster level, the average task-rest effect size is used to determine clusters; all edges surviving a cluster-determining threshold of |*d*| = 0.8 (i.e., edges with *d* > 0.8 and *d* < −0.8) are shown, and separate clusters of contiguous edges are counted. Likewise, all thresholds below |*d*| = 0.8 yielded only two clusters. Within-community connectivity, generally lower during task than rest, is highlighted by the yellow dotted rectangle. (*B*) Histogram of effect sizes at the edge level (40 bins), network level (20 bins), and whole-brain level (i.e., pooled across all edges; 2 bins). (*C*) Edge- and network-level effects by task. WM, working memory. (*D*) Variability between networks. Boxplots show the median (red line), interquartile range (IQR; blue box), and outliers (red whiskers; beyond 1.5 × IQR) of edges within each network. F-statistics quantify between- versus within-network variance, and significance is estimated by 1) shuffling node-community memberships (black), and 2) shuffling edges while keeping within- and between-community structure (green and orange). The vertical line separates within- from between-community networks, and the horizontal line indicates 0.

Several factors contribute to the ability to detect effects. The most important of these is the degree to which the spatial extent of ground truth effects matches the inferential procedure used. We estimated nonzero effects for all edges in the connectome, which correspond with the broadest scale of inference. To evaluate this decision, we examined the evidence against the null hypothesis across the connectome. On average across tasks, the majority of edges (87%, controlling FDR; 66%, controlling FWER) and networks (97%, FDR; 82%, FWER) showed significant differences between task and rest with a simple univariate contrast (*P* < 0.05, two-sided *t* test; average task effect sizes in Fig. 3*A* and *SI Appendix*, Fig. 3 *A* and *C*; effect size by task in Fig. 3*C*). Furthermore, clusters spanned the whole brain; only a single positive cluster and single negative cluster were found when using cluster-determining thresholds up to a large effect size (i.e., thresholds of |*d*| > 0.2, |*d*| > 0.5, and |*d*| > 0.8 each resulted in only two clusters; Fig. 3*A* and *SI Appendix*, Fig. 3*B*); only using a very large threshold (|*d*| > 1.0) produced more than two clusters in a very sparse graph (m = 2 positive clusters and m = 3 negative clusters). Pooling within networks or across the whole connectome increased effect sizes (Fig. 3*B* and *SI Appendix*, Fig. 3*D*), suggesting coordinated activity across widespread brain areas (58% of networks showed medium or larger effect sizes compared with 23% of edges). Importantly, the fact that the majority of effects estimated in the full sample are significant implies that estimated ground truth effect signs are meaningful, thus supporting the validity of their use for benchmarking power.

**Fig. 4.**
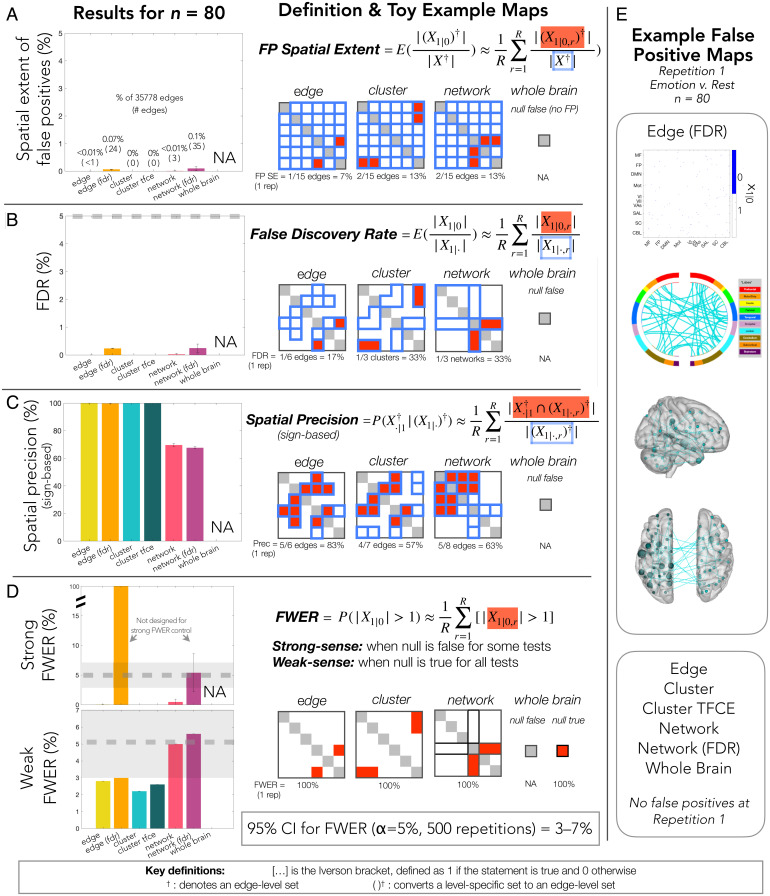
Specificity across levels of inference. Results from the following measures are shown at *n* = 80 averaged across all 7 tasks except as noted, alongside definitions and toy example maps (all sample sizes in *SI Appendix*, Fig. 9). (*A*) Spatial extent of false positives, defined as the proportion of the connectome showing false positives. (*B*) FDR, defined as the proportion of detections that are true positives. (*C*) Spatial precision, defined as the proportion of detections that overlap with ground truth effects in the same direction. (*D*) FWER, strong sense (*Top*) and weak sense (*Bottom*; obtained using the fake task contrast), defined as the percent of repetitions with at least one false positive. The expected 95% CI for FWER is highlighted in gray, and valid control is defined as falling below the upper bound. (*E*) Example false positive maps from the first repetition of emotion vs. rest shown via matrix, circle plot, and glass brain (no sparsification). Toy examples show results only for a single toy repetition and count only unique edges (e.g., lower triangle only). An abridged list of definitions for key terms and notation is provided at the bottom of the figure; for details, see *SI Appendix*, Fig. 1 and *Supplemental Method 4*.

However, we do not expect simple dependence across the whole connectome; networks also contributed unique information. The original Shen268 networks were significantly more heterogeneous than when randomly shuffling nodes across communities, suggesting that some information may be shared within each network that is not shared across all networks (Fig. 3*D*). Despite notable differences between within- and between-community effects, within-community edges were not interchangeable and neither were between-community edges; the original within- and between-community networks were more heterogeneous than when shuffling within- or between-community edges, respectively (Fig. 3*D*). The Shen268 partition also showed a fair amount of overlap with a partition defined in the HCP with the Louvain method (*SI Appendix*, Fig. 4). Thus, although pooling within the Shen268 networks is a fairly simple way to account for widespread dependence that is not refined by the structure of the data at hand, it captures some meaningful network-level structure in the independent HCP data (*SI Appendix*, *SI Results 2. Generalizability of the Shen268 partition to the HCP data* for details).

### How the Error Rate under Control (FDR vs. FWER) Influences Power.

As expected, power varied not only with scale of inference but also with the error rate under control, favoring FDR (although, also see *Cost in terms of specificity: False positives and localizing power*). Exploring this further, FDR-controlling procedures were more likely to detect an effect of a given size compared with FWER-controlling procedures (Fig. 2*D* and *SI Appendix*, Fig. 2*C*). In fact, controlling FDR instead of FWER was like having twice the subjects to detect the same sized effect—for example, for a medium-sized network-level effect, power with FDR control at 40 subjects was approximately equal to that of FWER control with 80 subjects (β_network_FDR,|_*_d_*_| = 0.5,_ *_n_*_= 40_ = 85%; β_network, |_*_d_*_| = 0.5,_ *_n_*_= 80_ = 84%). Of note, FDR-controlling procedures offered similar power for the same effect size regardless of level of inference. For example, edge (FDR) and network (FDR) approaches both had similar power to detect a medium-sized effect at *n* = 40 (i.e., for |*d*| = 0.5, *n* = 40: β_edge_FDR, |_*_d_*_| = 0.5,_ *_n_*_= 40_ = 80%; β_network_FDR, |_*_d_*_| = 0.5,_ *_n_*_= 40_ = 85%); yet, at the same time, there are far fewer medium-sized effects at the edge level than the network level (Fig. 3*B*). In contrast, there were clear differences between FWER-controlling procedures with an intermediate scale of inference actually showing the greatest benefit; cluster-level procedures offered the best power for a given effect size (especially cluster TFCE), whereas the edge-level procedure showed a disproportionately low power for the same effect size (e.g., for |*d*| = 0.5, *n* = 40: β_cluster_TFCE, |_*_d_*_| = 0.5, n = 40_ = 62%; β_cluster, |_*_d_*_| = 0.5, n = 40_ = 53% compared with β_edge, |_*_d_*_| = 0.5, n = 40_ = 3%).

### Spatial Bias in Effect Size and Power.

The spatial distribution of effects was strikingly consistent across tasks, with decreasing connectivity (toward zero) within-community and between motor and visual communities during task compared with rest (Fig. 3 *A* and *C* and *SI Appendix*, Figs. 5 and 6). These effects are therefore expected to be among the most readily detected during benchmarking. The consistency across task contrasts is primarily because, despite high similarity between task and rest connectomes, rest was distinct from each task in a consistent way. Differences between task and rest were further enhanced by the longer resting scan duration (*SI Appendix*, *SI Results 3. Differences between task and rest, and effect of unbalanced scan durations*).

It is already known that edges with larger effects tend to show greater power; we further explored whether some areas showed greater power independent of effect size by examining the residuals of the effect size-power curves (*SI Appendix*, Fig. 7). This revealed a spatial bias in power independent of effect size—that is, for the same effect size, effects were more likely to be detected in certain areas compared to others. Specifically, edge (FDR)- and cluster-level approaches were slightly more likely to detect effects in subcortical and cerebellar networks at small sample sizes, but this small bias decreased with the larger sample sizes.

### Cost in Terms of Specificity: False Positives and Localizing Power.

Researchers strive to detect as many effects as possible while simultaneously preventing the emergence of “too many” false positives. While how many is too many remains subjective, very few false positives (i.e., detections in the wrong direction) were observed relative to true positives. For each procedure, less than 0.5% of the connectome exhibited false positives (i.e., <179 edges; Fig. 4*A*), which constituted less than 1% of all detected effects (Fig. 4*B*). That is, one false positive was observed for at least every 100 true positives. As expected, FDR-controlling approaches were most permissive in this sense; FWER-controlling procedures had a much smaller FDR (below 0.25%). This same pattern was found for the weaker and sparser effect size ground truth maps but, as expected, more false positives were found for the sparser conditions (*SI Appendix*, Fig. 11). The sparsest condition showed the most false positives (up to 3.1% of the connectome), which is expected because it likely treats many nonneglible effects as null (in this condition, anything below a small effect size of *d* = 0.2 is null).

Another form of specificity important for researchers is spatial specificity—the ability to spatially pinpoint effects. By definition, broader scales of inference permit less certainty in localizing effects. In practice, we can estimate how much spatial precision is sacrificed by examining the extent to which detections overlap with true edge-level effects. Only edge- and cluster-level results showed perfect or near-perfect overlap with underlying edge-level effects (Fig. 4*C*). Yet, network-level procedures were not unreasonably imprecise—on average across repetitions, ∼70% of edges across all detected networks reflected true effects. Note that precision is not shown for the whole-brain procedure as it alone is based on a test of inequality as opposed to a one-sided test. Since all edges are nonzero in the ground truth, any significant result for the whole-brain procedure will necessarily have full spatial precision, but this is not directly comparable with the other procedures (*cf* *SI Appendix*, *Supplemental Method 4.5. Spatial Precision*). A similar pattern was observed for the weaker and sparser ground truth maps except that sparsity reduced spatial precision for all approaches, affecting the network-level FDR approach the most and the edge-level and cluster-level (Network-Based Statistic [NBS]) FWER approaches the least (*SI Appendix*, Fig. 11).

Finally, it is critical that all inferential procedures achieve the expected control of false positives. FWER-controlling procedures are designed to limit weak- and strong-sense FWER (i.e., when the null is true everywhere and not, respectively), whereas FDR-controlling procedures are designed to limit FDR (and, as a corollary, weak-sense FWER (18)). All procedures achieved valid control (Fig. 4 *B* and *D*), and no bias in the spatial distribution of false positives was observed (*SI Appendix*, Fig. 8). For the weaker and sparser conditions, only the sparsest experiment did not attain valid control for edge- and network-level approaches (*SI Appendix*, Fig. 11), which is again expected because it likely treats many nonneglible effects as null. There may be some room for improvement in edge- and cluster-level approaches, which appeared overconservative in controlling errors. Yet, the better power for broader scale inference was not solely due to relatively greater permissiveness; while network-level approaches permitted more errors, no false positives were observed for the whole-brain inferential approach despite its 100% power. No clear relationship was observed between sample size and FWER, although cluster- and network-level approaches were most conservative at the lowest sample sizes. Finally, the tradeoff between FDR and FWER control is readily apparent when examining strong-sense FWER results; at the edge level in particular, controlling FDR resulted in at least one false positive at every repetition, but this could be as small as one out of tens of thousands of edges.

## Discussion

Despite the popularity of focal inferential procedures, the present work empirically demonstrates that simple procedures that account for broad-scale dependence better reflect the spatial extent of effects and thus substantially improve power. Together with recent work in large and deep task-based activation datasets (3–5), these results indicate that cluster-based inference may only reveal the tip of the iceberg of true widespread effects in typically sized task-based studies. The present findings suggest that leveling up may be an optimal path forward for many typical studies that need to prioritize statistical power at the cost of a relatively modest decrease in specificity.

### Implications for the Scale of Functional Connectivity Inference.

Shifting to a broader level scope stands in contrast with the conventional focus on localization as the main priority. In fact, some have suggested that users consider employing more stringent cluster-determining thresholds to obtain even more focal inferences (19). However, the present results challenge the utility and meaningfulness of standard goals of focal inference. Broader scale procedures were better equipped to detect effects because they better matched the underlying widespread distribution of effects across a wide variety of tasks. This is consistent with the intuition and evidence that an organ as complex as the brain is unlikely to have areas wholly uninvolved in many studied cognitive processes (5, 20). In fact, increasing evidence suggests that key observable facets of brain processes operate in a low dimensional space (21). At the neuronal level, complex interactions between brain areas underlie emergent population-level properties that cannot be captured at the level of individual neurons; similar observations are becoming prevalent in neuroimaging (22). Notably, this substantial low dimensional structure is expected to account for the unmatched performance of the sole multivariate method tested here and in related recent work (14).

That said, there are certainly limitations to more diffuse approaches. While we found them to be beneficial across a variety of task-based contexts, they can gloss over unique information occurring in individual areas. In particular, approaches that combine information widely (most notably, the whole-brain procedure) can obscure the detection of truly focal effects in some contexts (e.g., pathology originating in a specific area) or hinder the discovery of focal targets for intervention (e.g., brain stimulation). Furthermore, broader scale procedures suffered a loss in spatial precision even when effects were widespread. However, the decrease in spatial precision seemed relatively modest compared with the gain in power, especially for typically sized studies using typical task paradigms. For example, given a sample size of 40 in a task-based study, which of the following would be preferable for a researcher: only 10% power but 100% spatial precision (as in edge FWER); quadruple the power (40%) and equal spatial precision but now controlling FDR instead of FWER to 5% (as in edge FDR); nearly double that power (75%) but with a modest loss in spatial precision (70%) (as in network FDR); or, finally, 100% power with a multivariate procedure but no localization to individual brain areas (as in whole brain)? This question is particularly poignant for typically sized studies, which may not be sensitive to focal effects even if they do occur in a study. Altogether, we believe that network-level inference, which balances aggregating widespread effects for power with retaining unique network-specific information for specificity, may be a step in the right direction for many typical studies.

### Matching Inferential Procedures to Spatially Extended and Multivariate Signals.

Many approaches can be used to capture distributed effects. One of the simplest options is to pool data within predefined areas in a mass univariate fashion, as in the Constrained NBC (cNBS) (11) approach used here. Simple pooling may be helpful if one expects pooled variables to be random realizations of a shared underlying effect (i.e., redundant) and noise to be relatively independent across variables within the pool (and thus averaged out). This can even be conducted more simply than the procedure presented here; if one is comfortable with the assumptions, one can eschew the nonparametric estimation of *P* values for a simpler parametric process involving averaging within edge groups, running a standard parametric test to estimate *P* values, and performing multiple comparison correction. Beyond this, there are many degrees of freedom, from the choice of the atlas and partition to the data aggregation strategy and more. A major concern is how to balance leveraging large datasets to inform smaller studies while also respecting the unique properties of the study at hand (e.g., study-specific network configurations (23)). Smaller studies stand to benefit the most from a priori partitions since they are most at risk for underestimating the spatial extent of effects. It can be challenging to choose from the many available resting-state network definitions to define this partition, but evidence suggests core components of these networks are fairly robust (24), so many definitions may be appropriate (one can also compare with the universal taxonomy in ref. 24). One can also remove networks from evaluation in a hypothesis-driven fashion to improve power; between-community networks may be good candidates for removal since more tests are conducted between- than within-community networks.

While mass univariate pooling within predefined areas has the advantage of simplicity and can be readily incorporated into research workflows, dependence in brain data is unlikely to be so simple. This may account for the lower performance in machine learning approaches that employ constrained pooling (25); such approaches likely combine complex multivariate information more effectively than pooling (although broader scale pooling (25) and summarization (26) can improve test–retest reliability for predictive models). One might choose to explicitly model dependence across the brain based on a priori expectations using a more principled approach (e.g., structural equation modeling, Bayesian analysis) or to estimate the dependence from the data itself and use it in a nested or (principled) circular (27) procedure. Note that broader scale inference does not preclude finer-grained analysis. In fact, the designers of cluster-level inference recommended that exploratory studies start with set-level inference and employ a step-down approach that should not increase FWER (1); this has recently been formalized with the All Resolutions Inference framework (28). Broader scale findings can also be used as a starting point to subsequently collect more data for more refined localization. It may also be valuable to explicitly incorporate information occurring at different levels simultaneously (20, 29). One may alternatively eschew localization altogether and leverage one of the many statistical and machine-learning approaches designed to capture the low dimensional, multivariate nature of the signal (e.g., manifold learning (22)).

Finally, as alluded to above, evidence increasingly points toward the trivial nature of a mass null hypothesis in the context of widespread, multivariate effects. One compelling option is to rethink traditional frequentist approaches and eschew estimates regarding the zero null in favor of those regarding effect size, as is done by the confidence sets approach (this also has the advantage of providing spatial extent estimates) (30). Alternatively, it may be useful to adopt a Bayesian approach for characterizing the full distribution of effects based on prior evidence (31, 32). Large openly available datasets may offer some insights for building these priors; more about the nature, structure, and limitations of the effects estimated here is discussed in *SI Appendix, SI Results 1. Limitations in generalizability of ground truth and performance measures*. Overall, the present results are just a demonstration and a starting point and merely scratch the surface of what possible inferential procedures can be used to account for dependence and combine information across the brain.

### Choosing FDR over FWER Control.

FDR-controlling procedures offered substantially greater power than FWER-controlling procedures, such that more focal inference when used with FDR actually outperformed broader scale inference when used with FWER. Neuroimaging researchers have been taught to prioritize the control of false positives and with good reason—as Richard Feynman famously said, “the first principle is that you must not fool yourself and you are the easiest person to fool.” Indeed, issues with the adequate control of false positives have haunted the field for quite some time, especially due to the need to correct for the large number of tests across the space of an fMRI image (17, 33). However, researchers pay relatively little attention to the fact that a more stringent control of false positives comes at the cost of greater false negatives (i.e., less power and true positives). Both types of error can meaningfully impair scientific discovery (34), and it may be prudent to instead prioritize a tradeoff between the two. FDR control is beneficial when one is willing to admit more false positives to obtain more true positives and should control FWER when the null is true everywhere (18). The resulting decrease in spatial specificity may be a reasonable sacrifice in the context of distributed fMRI effects in the present study.

The field has highlighted the potential power gains for FDR over FWER (35) (relatedly, see ref. 3), and recently, when the field reckoned with invalid FWER control for popular parametric cluster-level inferential procedures, a follow-up study suggested that results would still hold if studies had controlled FDR (36). Whether the lagging popularity of FDR is due to convention, the accessibility of tools, or other reasons, the present results underscore an opportunity for more FDR-based tools.

### Task Effects on Connectivity and (Limited) Generalizability to Other Contexts.

How the brain functionally reorganizes during task and rest is a major topic of study. As has been shown before (37), we observed that rest and task connectivity were highly similar. However, whereas paired differences between tasks were relatively unique, rest was distinct from each task in a consistent way; task connectivity was generally lower within communities and between motor and visual communities. Previous reports have also showed that integration is greater during task than rest (38, 39) and increases with cognitive demand (40), suggesting that more cognitively complex tasks require more collaboration across systems performing unique functions. These results together illustrate the divide between cognitively demanding task states and less demanding rest, which adds nuance to the common notion that rest explores the full repertoire of task-relevant states.

Since contrasting task with rest may reflect general demand-specific rather than cognition-specific effects, investigations may benefit from a more task-relevant reference—perhaps a task with similar cognitive demands, or an estimate of intrinsic connectivity that includes task (e.g., (41)). Importantly, task–rest differences were enhanced by the longer resting scan duration, and care should be taken to account for differences in the scan duration that can bias contrasts toward the longer (typically rest) scan. Besides being potentially misleading for drawing task-specific conclusions, rest data have also been shown to be suboptimal for predicting behavior compared with task data (42). As such, the role of rest in functional connectivity analysis bears careful consideration.

More generally, the spatial distribution and magnitude of effects are expected to vary substantially with study design, statistical models, covariates, and more. We aimed to test the robustness of the benchmarking results by not only including a variety of tasks but also evaluating weaker effect sizes and a sparser map. Yet, there are innumerable additional variations on the present study. Scan duration and trial number in particular are known to influence test–retest reliability (43, 44) and efficiency (45). The influence of these factors on study power remains to be determined. Brain–behavior associations in particular appear to be an order of magnitude smaller than the present task-based effects; perhaps due to weaker or more heterogeneous effects, differences between edge- and network-level results were not apparent for such associations (14). Thus, while we expect results to generalize to reasonably similar study designs, the spatial extent of effects and optimal level of inference for study designs that differ substantially are open questions (*SI Appendix*, *SI Results 1. Limitations in generalizability of ground truth and performance measures*).

### Implications for Data beyond Functional Connectivity.

This study is motivated in part by observations from task-based activation mapping; therefore, it is only fitting that we expect implications for that context as well. While many typically sized activation-based studies demonstrate results in localized blobs, larger and deeper studies show widespread activations across the brain (3–5). As such, a larger scale inference is expected to benefit activation-based studies as well. Yet, the best strategy for combining complex multivariate information in that context remains to be determined. Assigning areas to resting state communities may be a start, although this depends on the extent to which coactivation occurs within the bounds of these communities. Traditional dimension reduction procedures like independent component analysis may better reflect the coactivation patterns in the data at hand (46). While the methods that best capture this widespread signal remain an open question, there is growing appreciation in the community that localized processes are an artifact of small sample sizes and historical inferential procedures and only reflect the tip of the iceberg of true effects (3–5, 30). The present work adds indirect support to this perspective.

Outside of neuroimaging, much of biomedical research also struggles with maintaining power given high-dimensional, dependent data with small univariate effect sizes. Many other modalities in neuroscience are now being used to simultaneously record many signals (e.g., electroencephalography, optical imaging) and thus also reckon with spatially dependent observations. Genetic methodologists are also exploring approaches that capture widespread effects (47, 48) and translating methods to neuroimaging (12). Since we all face similar issues, it may be fruitful for neuroimaging to examine methods for evaluating and capturing dependence in other fields, and perhaps methods developed in neuroimaging may in turn have relevance to those contexts.

## Conclusion

Cluster-level inference enabled many important discoveries when the field was first exploring tailored procedures for more powerful inference beyond the voxel level. Today, larger-than-ever datasets and computational resources have facilitated the evolution of statistical procedures based on a more complete understanding of their accuracy and the nature of the signal. Here, we highlighted one important avenue toward continued improvement, as follows: the use of procedures designed to capture the widespread spatial effects across the brain, which provide a desperately needed boost in power for typically sized studies. More generally, the impressive rise in openly available datasets in the field presents an opportunity to better characterize elements of the signal to inform inference for more typical studies. As we learn more from the data, we grow better equipped to build tools tailored to the underlying signal, which, in turn, leads us closer to more robust and reproducible findings in neuroimaging.

## Methods

Functional connectivity data derived from the HCP S1200 release were used to estimate power and FWER. Minimally preprocessed data provided by the HCP were further processed to regress artifact and obtain connectivity matrices of the z-scored Pearson correlations between the 268 nodes of the Shen268 atlas (49).

A total of 168 experiments were used for benchmarking, encompassing 7 inferential procedures, 8 task contrasts, and 3 group sizes (*n* = 40, 80, 120). For each experiment, paired sample tests were used for 7 real task contrasts with rest (tasks = emotion, gambling, language, motor, relational, social, working memory; *n* = 1,021 to 1,058) or one fake task contrast (contrast between two resting runs, shuffled for each subject). At each of *R* = 500 repetitions, groups were resampled followed by inference.

The seven procedures used to perform inference at each of the four levels (edge, network, cluster, whole brain) included the following:

1) edge: a parametric procedure for FWER correction (Bonferroni procedure (50)), wherein a significant edge implies an effect at that edge,
2) edge (FDR): a parametric procedure for FDR correction (Storey procedure (51)), wherein a significant edge implies an effect at that edge,
3) cluster: the NBS method for cluster-level inference in the connectome with permutation-based FWER correction (2), wherein a significant cluster implies an effect for at least one edge within that cluster,
4) cluster (TFCE): threshold-free NBS, a threshold-free method for cluster-level inference with permutation-based FWER correction (52), wherein a significant edge implies an effect for at least one edge in all clusters associated with the significant edge,
5) network: the cNBS method we recently introduced for network-level inference (11) with permutation-based estimation of network-level nulls followed by parametric FWER correction (Bonferroni procedure), wherein a significant network implies an effect for the pooled network (but not for any edges in particular within that network),
6) network (FDR): the cNBS method with FDR correction (Simes procedure (53)), wherein a significant network implies an effect for the pooled network (but not for any edges in particular within that network), and
7) whole brain: the multivariate cNBS statistic (mv-cNBS) we introduce here for whole-brain multivariate inference based on cNBS, wherein a significant test implies a multivariate effect based on pooled networks.

This included both a standard procedure and a procedure expected to be more powerful for each level except the whole brain, for which a single procedure was used. Null distributions were estimated nonparametrically (i.e., with permutation) for all but the edge-level procedures, which used parametric approaches for feasibility (*SI Appendix*, *SI Results 4. Nonparametric edge-level FDR inference implemented in the NBS toolbox*). One-sided tests were used for all but the whole-brain procedure, which relied on a single multivariate statistic. The Shen268 atlas was also used to define 10 node communities (*cf* ref. 54) for partitioning the graph for cNBS and mv-cNBS. All inferential procedures have been implemented as extensions to the Matlab *NBS* toolbox (except the NBS procedure, for which we used the original procedure implemented in the toolbox). The toolbox was extended for use in the Matlab command line.

For each resampling repetition, true positives were defined as effects detected during resampling for the real task contrast found in the same direction as the ground truth effect from the full sample, analogous to one-sided tests conducted in the correct direction. False positives were defined as effects found in the opposite direction as the ground truth effect for the real task contrast or any effects found during the fake task contrast. Six measures of accuracy were then estimated to explore the balance between true and false positives, as follows: power, spatial extent of false positives, false discovery rate, spatial precision, strong-sense FWER, and weak-sense FWER (i.e., when the null is true everywhere; this is the only measure that used the fake task contrast).

## Supplementary Material

Supplementary File

## Data Availability

All code for inference, benchmarking, and summarization used here is available at: https://github.com/SNeuroble/NBS_benchmarking (55). See *SI Appendix, Supplemental Methods* for details. Publicly available data were used for this work (56).

## Footnotes

^*^When mapping brain areas, researchers typically estimate a statistic at each of the smallest units of the brain scan, called voxels, and then perform inference by pooling information across clusters of neighboring voxels. This is more powerful than voxel-level inference when neighboring voxels show sufficient dependence (i.e., share similar properties)—as is the case in fMRI^1^—in part because it avoids multiple testing correction across thousands of relatively noisy voxels. Cluster-based procedures have also been translated for inference in fMRI brain networks or functional connectivity; namely, the NBS^2^ is used to perform inference on clusters defined as groups of adjacent edges (i.e., components).
