# Supplementary material for: Improving power in functional magnetic resonance imaging by moving beyond cluster-level inference
Source: Proc Natl Acad Sci U S A. 2022 Aug 4;119(32):e2203020119. doi: 10.1073/pnas.2203020119 (PMC9371642; doi:10.1073/pnas.2203020119)
Supplement: Supplementary File [file pnas.2203020119.sapp.pdf]

SUPPLEMENTAL METHODS

The following details the inferential procedures under evaluation (**SI Methods 1**), data used and preprocessing strategy (**SI Methods 2**), benchmarking procedure (**SI Methods 3**), and estimation of accuracy metrics (**SI Methods 4**). All code was written in Matlab (see **SI Methods 5** for resource availability and technical details). To the best of our knowledge, the reporting in this manuscript was consistent with the guidelines provided by the Committee on Best Practice in Data Analysis And Sharing (COBIDAS; see **SI Methods 6** for details).

1. Description of Inferential Procedures

Seven procedures were used to perform inference at each of the four levels, referred to here as "edge", "edge (FDR)", "cluster size", "cluster TFCE, "network", "network (FDR)", and "whole brain". All procedures rely on a user-specified GLM to estimate univariate (edge-level) statistics and specify a target familywise error rate (FWER) or false discovery rate (FDR) of 5%. All procedures are nonparametric except the edge-level procedures and use K=1,000 permutations to estimate the null using the procedure implemented in the *NBS* toolbox (1). For each permutation, rest and task data are randomly exchanged for each subject, edge-level statistics are calculated, then the null statistic for the given inferential procedure is estimated. This procedure has been shown to offer weak but exact (on average) control of the desired FWER level with a per-repetition 95% confidence interval corresponding with K=1,000 of FWER = 0.0500 ± 0.0138 (https://fsl.fmrib.ox.ac.uk/fsl/fslwiki/Randomise/Theory).

All procedures were implemented for the present study as extensions to the Matlab *NBS* toolbox except the "cluster" (NBS) procedure, which used the procedure originally implemented in the toolbox. The toolbox was also modified to be run from the Matlab command line rather than as a toolbox-specific GUI.

1.1. Edge Procedure

The "edge" procedure used the Bonferroni algorithm to correct edge-level p-values. The parametric Bonferroni procedure is intended to control the FWER to a specified level and has been previously described in detail by (2).

1.2. Edge (FDR) Procedure

The "edge (FDR)" procedure used the Storey algorithm as implemented in the *mafdr* tool to correct edge-level p-values. The parametric Storey procedure is intended to control the FDR to a specified level and has been previously described in detail by (3). Note that the FDR is expected to control the FWER at the specified level when all tests are drawn from the null (4).

1.3. Cluster Size Procedure

The "cluster size" procedure used the Network-Based Statistic (NBS) algorithm implemented in the *NBS* toolbox version 1.2 ((1); https://www.nitrc.org/projects/nbs). In brief, at each of K permutations, a cluster-determining threshold (here, *t*=3.1, corresponding with *p*=0.005-0.001 for DOF=10-1,000) is applied and each group of contiguous edges (i.e., edges that are connected by a node) is identified as a separate cluster (i.e., "component"). The present study used cluster size as the target statistic, which reflects the number of contiguous edges in a cluster. The maximum cluster size is then recorded at each permutation, and this set of maxima forms the null for inference. That is, significant target (unpermuted) data clusters are determined by comparing the sizes of all target clusters with the cluster sizes in the null.

As an alternative to cluster size, one may also use cluster intensity as the target statistic. Both are implemented in the *NBS* toolbox. Cluster intensity uses the sum of the edge-level statistics within the cluster as the target statistic. Preliminary testing yielded very similar results, so size was chosen for all NBS analyses presented in this study.

1.4. Cluster TFCE Procedure

The "cluster TFCE" procedure implemented here is an analogue of threshold-free cluster enhancement in the task-based activation literature (TFCE; (5)) and echoes other TFCE methods for NBS described in the literature (6, 7). This procedure follows the cluster size procedure described above except the TFCE statistic is used as the target statistic. The TFCE statistic is defined for each edge *x* as:

$$TFCE(x) = \int_{h=h_0}^{h_x} e(h)^E \, h^H \, dh$$

where *h* is the magnitude (i.e., strength) of the edge-level statistic (h_0 is typically zero and h_x is the magnitude at edge *x*), *e* is the cluster extent of all surrounding edges ≥ *h*, and *E* and *H* are constant weighting parameters for *e* and *h.* Put another way, the TFCE statistic for an edge is essentially a

weighted sum of surrounding edges in the cluster containing that edge, where clusters are defined by a CDT of 0 and the tops of nearby peaks are discounted from the sum. In contrast, inference based on cluster size relies on the user to set the minimum meaningful effect size, which can lead to results that are arbitrarily threshold-dependent.

As in the activation context (5), the values of E and H are determined empirically. The present study implemented TFCE and used the default parameters of H=3 and E=0.4 determined for the *Mrtrix* software ((8); see https://github.com/MRtrix3/mrtrix3/blob/master/cmd/connectomestats.cpp) based on (9), although the developers caution that the optimization of these parameters may not be precise and that results are not robust to small perturbations in the parameters. That said, these parameters are also close to one set of values recommended by in ((6); H=[2.25-3] and E=0.5; the other recommended set is H=[3-3.5] and E=0.75), although that study suggests suboptimal power with E<0.5 so the present value of E may be suboptimal.

While the other levels of inference controlled FDR as the “better” procedure, to our knowledge there is not an available nonparametric procedure for controlling cluster-level FDR. Thus we selected the TFCE procedure as the “better” cluster-level procedure since it has been previously demonstrated to improve power compared with cluster size.

1.5. Network Procedure

The “network” procedure used the Constrained Network-Based Statistic (cNBS) algorithm we previously implemented as an extension to the *NBS* toolbox (10). The algorithm is described in detail in (10) and reproduced in **SI Fig. 13**. In brief, cNBS aggregates edge-level statistics within predefined networks rather than using clusters defined by the data at hand, estimates the null for each network via permutation, then uses a parametric algorithm to control a specified false positive rate. The “network” procedure in this section used Bonferroni correction to control the FWER. Thus cNBS is a mixed parameteric-nonparametric approach.

The present study used the Shen268 268-node, 10-community partition defined using the Yale High-Resolution Controls dataset (n=40; (11); https://fcon_1000.projects.nitrc.org/indi/retro/yale_hires.html). Just as voxels were grouped into 268 nodes using the Normalized Cut procedure as described previously (11), this partition was created by grouping nodes into 10 communities using the Normalized Cut procedure, without requiring nodes within a community to be spatially contiguous. The 10 communities of nodes are used to define 10 *i* 11 / 2 = 55 unique networks representing connections within and between all communities; these 55 networks are used in cNBS for inference.

1.6. Network (FDR) Procedure

The "network (FDR)" procedure used the same cNBS algorithm as above, except the Simes procedure (12) (developed for FDR control by Benjamini-Hochberg; (4)) was used to control FDR (cf. **SI Fig. 13**). Note that "network FDR" uses a different FDR controlling algorithm than "edge FDR", which used the Storey algorithm. While the Storey algorithm is expected to be more powerful (3), the Simes algorithm was chosen for network FDR because it is faster as it does not use bootstrapping to estimate the null, and some have suggested it may yield nearly equivalent results as the Storey algorithm in practice (13).

1.7. Whole Brain Procedure

The "whole brain" procedure used mv-cNBS, introduced for the first time here as a multivariate analogue of cNBS that integrates information across the whole brain (cf. **SI Fig. 13**). Mv-cNBS uses the cNBS statistic vector *s* as the test statistic. The distribution of null vectors is then estimated via permutation. Finally, deviations of null vectors from the null centroid as measured by Euclidean distance are compared with the deviation of target-statistic vector from the null centroid to get a p-value. Since the full vector is used as a test statistic this procedure does not require multiple testing correction.

We introduce a couple considerations for the use of mv-cNBS. Mv-cNBS can be thought of as a form of omnibus test (i.e., indicating whether there is a departure from the null at at least one of the network-level test statistics). One implicit assumption is that all variables have equal variance and no covariance, such that the same magnitude test statistic excursion in any direction will result in the same p-value and all variables contribute equally to the variance of the test statistic. This is unlikely strictly true; an important example is that smaller networks may be more variable since they average over fewer edges. While we assume this is not a problematic assumption, it may be helpful to estimate covariance (from factors such as network size or the permutation-based null) for whitening.

Second, the null centroid is currently used as the reference for estimating distance. Re-centering at the null is not done in a univariate permutation test (e.g., a reference of zero is used for one-sample tests). While re-centering would not affect p-value estimates for a univariate test since rankings would remain the same, it can affect rankings for this multivariate test (i.e., the smallest data point in the ranking will be that closest to the reference data point, which could change substantially with choice of reference). The most important flaw with this definition is that if all null data points are sufficiently far from zero, then a zero test statistic (e.g., no effect at any network) could be seen as significant. In

practice, sufficient permutations should yield a centroid close to zero, although this can decrease with more variables and fewer repetitions. Note that we were able to achieve expected control of FWER, suggesting that the limitations discussed above may not lead to inflated false positives at least when the null is true everywhere.

2. Data and preprocessing strategy

An overview of key data characteristics and benchmarking parameters are given in **SI Table 1**.

2.1. Data description

The present study used data from the Human Connectome Project S1200 release (14). Data acquisition parameters have been described in detail elsewhere (15); in brief functional data were acquired with a slice-accelerated multiband gradient-echo echo planar imaging (EPI) sequence on a 3T Siemens Skyra (TR=720 ms, TE=33.1 ms, flip angle=52 degrees, resolution=2 mm^3, multiband factor=8). All available task data in volume space were included. No exclusion criteria were used and data were not stratified by family (see **SI Results 1. Limitations in generalizability of ground truth and performance measures**), although participants who did not have both scan conditions for a given paired sample contrast (e.g., both EMOTION and REST1 data for EMOTION-REST1) could not be selected for that contrast. No task regression was used; this can be thought of as retaining task activation-specific effects (16). The present use of publicly available, de-identified data from the Human Connectome Project and sharing of analysis results has been reviewed and designated as exempt (Exemption 4) by the Yale University Institutional Review Board.

**Category**	**Description**
Dataset	Human Connectome Project S1200 release (N = 1200 subjects)
Resampling sample size	n = 40; n = 80; n = 120
Tasks	**Tasks used for the 7 real task contrasts (task-versus-REST1):**<br>EMOTION (N=1022; 176 frames) — shortest task<br>GAMBLING (N =1057; 253 frames)<br>LANGUAGE (N =1021; 316 frames)

	MOTOR (N =1058; 284 frames)<br>RELATIONAL (N =1016; 232 frames)<br>SOCIAL (N =1027; 274 frames)<br>WORKING MEMORY (WM; N =1058; 405 frames) — longest task<br>REST1 (N =1067; 1200 frames)<br><br>**Tasks used for the 1 “fake task” contrast (REST1-versus-REST2, shuffling labels for each subject at each repetition):**<br>REST1 (N =1067; 1200 frames)<br>REST2 (N =1011; 1200 frames)
Resampling repetitions	R = 500
Test type	Paired-sample, one-sided
Inferential procedure	**edge:** Bonferroni procedure for FWER control (parametric)<br>**edge (fdr):** Storey procedure for FDR control (parametric)<br>**cluster:** Network-Based Statistic (NBS) procedure with FWER control<br>**cluster tfce:** Threshold-Free Cluster Enhancement NBS (tfceNBS) procedure with permutation-based FWER control<br>**network:** Constrained NBS (cNBS) procedure with mixed nonparametric-parametric (Bonferroni) FWER control<br>**network (fdr):** Constrained NBS (cNBS) procedure with mixed nonparametric-parametric (Storey) FDR control<br>**whole brain:** Multivariate cNBS (mv-cNBS) procedure (does not require multiple testing correction)

Permutations for nonparametric procedures	K = 1000

Supplemental Table 1. Key methodological details. Data characteristics and benchmarking parameters.

2.2. Preprocessing and first-level analysis

Minimally preprocessed data released by the HCP team were used for estimating connectivity. The minimal preprocessing pipeline has been described previously (17, 18); in brief, this included "gradient unwarping, motion correction, fieldmap-based EPI distortion correction, brain-boundary-based registration of EPI to structural T1-weighted scan, non-linear (FNIRT) registration into MNI152 space, and grand-mean intensity normalization," spatial smoothing with an "unconstrained 3D Gaussian kernel of FWHM=4mm," computation of activity estimates from the general linear model (including corrections and confound modeling), then temporal filtration and prewhitening.

Data were then further preprocessed in volume space using legacy BioImage Suite (BIS (19); https://medicine.yale.edu/bioimaging/suite/; the modern version of the software is BISWeb; https://bioimagesuiteweb.github.io/webapp/). Noise covariates were regressed from the data, including linear, quadratic, and cubic drift, mean white matter signal and cerebrospinal fluid signal (defined by automatic segmentation), and a 24-parameter model of motion (six rigid-body motion parameters, six temporal derivatives, and squared terms; (20)). No volume censoring was used. A Gaussian spatial filter was applied (approximate cutoff frequency=0.12Hz). Connectivity matrices were then calculated via Pearson's correlation between all mean node timecourses in the Shen268 atlas, followed by Fisher transformation to *z*-scores. Finally, left-to-right and right-to-left encoding connectivity matrices, acquired in subsequent runs for each scan condition, were averaged together for each subject. To perform a follow-up analysis about how scan duration influenced task-rest contrasts, connectivity matrices were also estimated from "trimmed" REST1 data, which matched the scan duration of the shortest task (176 frames in the EMOTION task).

3. Benchmarking performance

The present study used the benchmarking procedure we previously introduced in (10) (see for algorithm and details); this builds on our previous work (21) and was inspired by (22, 23). As in (10), resampled data were compared with the full sample "ground truth" dataset to determine whether

effects were detected in the direction dictated by the ground truth effect sign. Each test estimated changes in connectivity due to a specified task compared with rest.

3.1. Estimation of ground truth effect sizes for each task

For the "real" task contrast, paired sample contrasts were performed for the full sample comparing each task versus rest (Rest1; *N*=1016–1058). There are many ways to estimate task-related effects in functional connectivity; here, we use a simple context independent analysis (i.e., original timeseries without using a task regressor), which may be more phenotypically relevant than a context-dependent analysis (i.e., based on a task regressor; (24)). Edge-level effect sizes were calculated directly from the edge-level *z*-scores for each subject, which represent standardized edge strength. Network-level effect sizes were calculated by first averaging *z*-scores within networks for each subject. Similarly, whole brain-level effect sizes were calculated by averaging *z*-scores across the whole connectome for each subject. Paired one-sample *t*-statistics were then obtained for each variable by fitting a GLM with the *NBS* toolbox. *T*-statistics were then converted to Cohen's *d* coefficients used to measure effect size here. The conversion for a one-sample *t*-statistic is $d_s = \frac{t}{\sqrt{n}}$, where d_s is the Cohen's *d* coefficient for the sample, *t* is the *t*-test statistic, and *N* is the sample size (from eqn. 2.5.9. of Cohen, 1988, pp. 72; cf. (21)). For reference, effect size is frequently classified as small *d*=0.2, medium *d*=0.5, and large *d*=0.8 (25).

A follow-up analysis was conducted to perform benchmarking in the context of weaker ground truth effect sizes. This analysis followed the above procedure but contrasted the Emotion task with the Gambling task.

For the "fake" task contrast, all "ground truth" effect sizes are defined to be 0 (i.e., the null hypothesis is true at all edges).

Note that the whole brain-level effect size (pooled across all connectome edges) does not match the statistic used to perform whole brain-level inference (Euclidean distance of cNBS vector from the null centroid). While one option may have been to use Mahalanobis distance as the multivariate generalization of Cohen's *d*, Mahalanobis distance cannot be directly interpreted like Cohen's *d* (i.e., with rules of thumb for small, medium, and large) and thus we opted for a more interpretable pooled effect size. The whole brain-level effect size is not used in subsequent calculation of accuracy metrics.

Finally, there are important limitations on the interpretation of these ground truth estimates; see a detailed discussion in **SI Results 1. Limitations in generalizability of ground truth and performance measures**.

3.2. Estimation of detections via resampling

Data were resampled (i.e., repeatedly subsampled) at each of R=500 repetitions. For each repetition, n=40, 80, or 120 subjects were randomly selected without replacement from the full dataset of N subjects for group-level analysis.

Resampling was conducted across three group sizes chosen to span from “small” to “high” sample sizes compared with typical sample sizes for this field (26, 27). The smallest sample size is higher than typical for the field yet still only just adequately powered (power=80%) to detect a medium-sized effect when using a single uncorrected one-sided t-test (27), and the largest sample size is much larger than typical (27) but again only just adequately powered to detect a small effect when using a single test. Resampling was also conducted for eight different scan contrasts (seven real task contrasts and one “fake” task contrast). For the “fake” task contrast, when subsampling a group of subjects for benchmarking, the REST1 and REST2 scans were randomly shuffled for each subject. The idea of using shuffled regressors in resting state data to define a fake task contrast where all positives are false was inspired by (23).

Each inferential procedure was used to perform paired one-sample, one-sided tests resulting in a map of detections (i.e., significant tests). This procedure was repeated switching the sign of the tail used for the one-sided test to record detections across repetitions for the positive and negative tail in two separate maps. The real task contrast records were used to calculate all accuracy metrics (**SI Methods 4**) except weak FWER, which used the “fake” task contrast (**SI Methods 4.5**).

4. Estimation of accuracy metrics

The following statistical terminology defining true and false positive counts and accuracy metrics are derived in part from terminology and notation in (28), which we used previously (21). Here, X refers to a set of edges, clusters, networks, or a single whole brain variable. The decision to use set notation rather than the count notation (i.e., where X is a count) used in those previous works permits us additional flexibility in denoting individual members of the set, reserving notation for the cardinality of the set $|X|$ for counting.

The subset of X exhibiting a positive (a=1) or no (a=0) detection given a true (b=1) or no true (b=0) effect is denoted as

$$X_{a|b} = X_{\text{detection} \mid \text{true effect}} = X_{\text{null rejected} \mid \text{null false}} \subset X \ .$$

a = · or b = · denotes all conditions (e.g., $X_{\cdot|1}$ is the set of all true effects regardless of detection).

Special notation is used to designate edge-level sets required for some accuracy metrics. Edge-level sets are denoted with † (e.g., $X^{\dagger}$), and ( )† is used to designate a level-specific set that has been converted to an edge-level set (e.g., $(X_{1|0})^{\dagger}$). For reference, this notation is used to define several quantities that appear in the following sections, including:

- $X^{\dagger}$: the set of all edges ,
- ${X_{a|b}}^{\dagger}$: the edge-level set $X_{a|b}$, and
- $(X_{a|b})^{\dagger}$: the set of edges in a level-specific set $X_{a|b}$.

A graphical depiction of the following accuracy metrics can be found in **SI Fig. 1**.

4.1. True and false positives

True and false positives were estimated by comparing detected tests to "ground truth" effects for each repetition. In detail, the following procedure was used for edge- and network-level inference. The sign of the ground truth effect at each edge or network x in X was determined from the real task contrasts. The null was considered false ($x_{\cdot|1}$) for positive-tailed tests if the effect sign was positive, and for negative-tailed tests if the effect sign was negative. When the test direction did not match the effect sign, the null was considered true ($x_{\cdot|0}$). Thus, if x was detected ($x_{1|\cdot}$) for a test in the same direction as the ground truth sign, it was considered a true positive ($x_{1|1}$). A detection for a test in the opposite direction as the ground truth effect sign (in the real task contrast) or for which the ground truth was null (in the fake task contrast) was considered a false positive ($x_{1|0}$). Note that the real task contrast was mainly used for calculating false positives; the fake task contrast was only used for calculating false positives in the context of weak-sense FWER. Only positive tail results were used for the fake task contrast, since the permutation is expected to produce symmetric test results.

For cluster-level inference, a true positive cluster ($x_{1|1}$) is defined as a cluster where the null is false for at least one constituent edge in that cluster (${x_{\cdot|1}}^{\dagger}$), using the same condition of congruent test direction and effect sign to define where the null is false. A false positive cluster occurs when the null is true at all constituent edges (${x_{\cdot|0}}^{\dagger}$). Note that rejecting the null for a cluster only implies that the null is false for at least one edge in that cluster but does not specify for which edge(s) this is the case, and

rejecting the null for an edge based on the TFCE statistic implies that the null is rejected for some cluster containing that edge (5).

For whole brain-level inference, only the positive tail results were used for task-based benchmarking and any detection from the real task contrast was considered a true positive ($x_{1|1}$). This is because the test statistic uses only the magnitude of the distance from the null centroid and thus is always positive for the non-zero effects. Thus, false positives ($x_{1|0}$) only existed for the whole-brain procedure in the fake task contrast.

A follow-up analysis was conducted to perform benchmarking in the context of a sparser ground truth map. This analysis followed the above procedure but defined all effects less than a given threshold (d=0.1, d=0.2) as null and detections in either direction were considered false positives. All specificity metrics were calculated for both tails separately and then averaged together.

4.2. Power

Power reflects the ability to detect a known effect. For each edge, network, or whole brain statistic *x*, power was estimated as the number of times *x* was detected in the same direction as its effect sign ($x_{1|1}$) across all resampling repetitions divided by the total number of repetitions (R):

$$Power(x) = P([x_{\cdot|1} \in X_{1|\cdot}]) \approx True\ Positive\ Rate(x) = \frac{1}{R}\sum_{r=1}^{R}[x_{\cdot|1} \in X_{1|\cdot,r}]$$

where [...] is the Iverson bracket (defined as 1 if the statement is true and 0 otherwise).

Cluster-level inference was the only inferential procedure for which the spatial map of power was calculated at a different spatial scale than the level of inference—namely, at the edge-level. This is because cluster-level inference has edge- and not cluster-level implications, and thus it makes more sense to ask “how often do we detect this edge as part of a cluster” than “how often do we detect this cluster”. Classifying cluster-level detections as true or false requires knowledge of the null at the edge-level; a cluster is defined as a true positive if there is at least one constituent edge where the null is false, and a TFCE statistic is defined as a true positive if the null is false for some cluster containing that edge (5). In contrast, rejecting the null for a cluster does not imply that the null is false for any particular edge in that cluster. In the activation-mapping context, others have synonymously interpreted a voxel belonging to a detected cluster as a true positive when the null is false for an voxel (5, 21, 29).

To determine the true positive rate for cluster-level inference with respect to an individual edge ($x^{\dagger}$), we can determine the number of times it was part of a cluster that was detected in the same direction

as its edge-level effect sign; for a given direction, this is the number of times an edge showing a true effect ($x^{\dagger}_{\cdot|1}$) could be found within the edges of a detected cluster ($(X_{1|\cdot})^{\dagger}$). This is then divided by the total number of repetitions:

$$For\ cluster-level\ inference:\quad Power(x^{\dagger}) = P\left(\left[x^{\dagger}_{\cdot|1} \in (X_{1|\cdot})^{\dagger}\right]\right) \approx \frac{1}{R}\sum_{r=1}^{R}\left[x^{\dagger}_{\cdot|1} \in (X_{1|\cdot,r})^{\dagger}\right]$$

4.3. Spatial extent of false positives

The *spatial extent of false positives* reflects the size of false positives. It was estimated by mapping false positive detections to the edge level and counting the proportion of those edges in the connectome across repetitions:

$$False\ Positive\ Spatial\ Extent = E\left(\frac{|(X_{1|0})^{\dagger}|}{|X^{\dagger}|}\right) \approx \frac{1}{R}\sum_{r=1}^{R}\frac{|(X_{1|0,r})^{\dagger}|}{|X^{\dagger}|}$$

4.4. False discovery rate (FDR)

The *false discovery rate* reflects the tradeoff between false and true discoveries. False discovery rate was estimated by counting the proportion of all detections that were false detections across repetitions:

$$FDR = E\left(\frac{|X_{1|0}|}{|X_{1|\cdot}|}\right) \approx \frac{1}{R}\sum_{r=1}^{R}\frac{|X_{1|0,r}|}{|X_{1|\cdot,r}|}$$

4.5. Spatial precision

A major concern in neuroimaging is the localizing power of a procedure, or the ability to pinpoint where in a map an effect occurred given detections from that procedure (cf. (30)). A formal definition does not appear to be readily available in the literature; we therefore introduce the following as one possible measure.

Here, we define *spatial precision* as the extent to which a map of results contains true effects occurring at the smallest level of interest (the edge level in the present study), intended to reflect localizing power of any detections relative to the true extent of effects at the smallest level of interest. We distinguish this from *theoretical localizing potential* relative to the smallest level of interest, which we instead define as the extent to which a method may theoretically be expected to overlap with a single effect occurring at the smallest level of interest and reflects the uncertainty in the exact location of effects (e.g., here, a network of size 100 edges may have theoretical localizing potential relative to the edge level of 1 edge/100 edges = 1%).

Spatial precision was estimated by mapping detections to the edge level and counting the proportion of detections that were true detections across repetitions:

$$Spatial\ precision = P(\ X_{\cdot|1}^{\dagger}\ |\ (X_{1|\cdot})^{\dagger}\ )\ \approx \frac{1}{R}\sum_{r=1}^{R}\frac{|\ X_{\cdot|1}^{\dagger}\ \cap (X_{1|\cdot,r})^{\dagger}\ |}{|(X_{1|\cdot,r})^{\dagger}|}$$

Spatial precision can be thought of as akin to "positive predictive value" (PPV) in that it reflects the proportion of the ground truth effects that are detected. We refrain from calling this PPV since it does not reflect a "real" true positive rate. A "real" true positive should be interpreted at the level it was measured (e.g., network for network); since spatial precision seeks to provide insight into finer levels than actually measured (e.g., network to edge), we use the phrase "spatial precision" to disambiguate this term. We also note that this metric is similar to the Dice and Jaccard coefficients in that all three can be used to measure the intersection between edge-converted detections and ground truth maps; the difference is that Dice and Jaccard measure the extent to which the edge-converted detections and ground truth maps perfectly overlap whereas spatial precision measures the extent to which edge-converted detections reflect true effects.

To facilitate a fair comparison, the main text only reports spatial precision for one-tailed procedures, which excludes the whole-brain procedure. Unlike the other procedures for which a significant test implies a false one-tailed null (i.e., an effect in one signed direction), a significant test for the whole brain procedure implies the two-tailed null is false (i.e., an effect in either direction) for at least one edge. Since the ground truth definition implies that the two-tailed null is false for all edges, any significant result for the whole brain procedure will necessarily have full spatial precision. Yet reporting full spatial precision for the whole-brain procedure can be misleading for two reasons: 1) it is the only procedure for which there is no chance of achieving less than full spatial precision, 2) unlike other procedures, it lacks the dimension of precision that other procedures have in that it does not carry information about the effect sign. One may alternatively be interested in quantifying other ways in which the detected effect matches the underlying ground truth effect, for example, by measuring the similarity between the magnitude and sign of the estimated constituent statistics and the ground truth effects.

4.5. Familywise error rate, strong and weak

Familywise error rate (FWER) was estimated by counting the number of repetitions for which at least one false positive was detected divided by the number of repetitions with at least one true null:

$$FWER = P(|X_{1|0}| > 1) \approx \frac{1}{R}\sum_{r=1}^{\mathrm{R}}[|X_{1|0,r}| > 1]$$

where $|X_{1|0}|$ is the total number of false positive edges, clusters, networks false positives or a single false positive whole brain test. This value is divided by the total number of repetitions (*R*) since all repetitions contained at least one true null ($|X_{\cdot|0}| > 1$).

The real task contrast was used to estimate strong-sense FWER and the fake task contrast (i.e., true null everywhere) was used to estimate weak-sense FWER. Estimation of weak-sense FWER is the only context for which the fake task contrast was used. Note that there are no true nulls for the whole-brain procedure during the real task contrast, so strong-sense FWER is not calculated in that context.

5. Resource availability and technical details

Scripts for performing all inferential procedures, benchmarking, summarization, and visualization in Matlab are available at https://github.com/SNeuroble/NBS_benchmarking. Altogether, 7 inferential procedures, 8 contrasts, and 3 resampling group sizes were run for benchmarking power and FWER, resulting in a total of 168 experiments. Benchmarking was run on the Farnam High Performance Computing cluster at Yale via the Simple Linux Utility for Resource Management (SLURM) workload manager (https://docs.ycrc.yale.edu/clusters-at-yale/clusters/farnam/). Requested resources per experiment included 13 CPUs and 40GB memory on compute nodes from the general partition (Red Hat Enterprise Linux Server 7.9). Repetitions were parallelized within each experiment using Matlab *parfor*. The following were the average run times across tasks for individual experiments (13 parallel jobs, R=500 repetitions and K=1,000 permutations per job): [0.2, 0.4, 0.6] hours for edge, [0.3, 0.5, 0.7] hours for edge FDR, [3.2, 6.7, 11.4] hours for cluster, [6.9, 8.9, 12.7] hours for cluster TFCE, [3.1, 6.2, 10.1] hours for network, [3.2, 8.0, 10.0] hours for network FDR, and [3.1, NA, 11.2] hours for whole brain (intervals represent run time corresponding with for group size *n*=40, *n*=80, *n*=120; note that *n*=80 for whole brain took longer than a day to run for unexplained reasons so that run time is left as NA). For comparison, sequential run time for a single experiment is estimated to be as little as 2.6 hours (40 subjects, edge) or as much as 165 hours (120 subjects, cluster TFCE).

In addition to the above, experiments were also run to benchmark nonparametric FDR as implemented in the *NBS* toolbox. By far, nonparametric FDR took the most resources to run, requiring double to triple the memory and time to run as the other approaches. Since FWER control was not achieved with the above number of permutations, nonparametric FDR experiments were also repeated with 10,000 permutations but FWER was still not achieved and we discontinued this evaluation.

6. COBIDAS compliance

To the best of our knowledge, the present manuscript is compliant with the mandatory COBIDAS recommendations (i.e., those marked Y in the checklist). The following summarizes which categories have and have not been explicitly described in the present manuscript as well as our rationale.

Data description categories that were described in the present manuscript include: Number of subjects (excluded and analyzed), Ethical considerations (for local IRB), Power analysis, Preprocessing reporting (further processing beyond HCP minimal pipeline), Statistical Modeling and Inference: Mass univariate analyses, Functional connectivity, and Multivariate modeling, Results reporting: Mass univariate analysis, Data Sharing, and Reproducibility.

A number of categories were not described in the present manuscript because they have been previously extensively documented by the Human Connectome Project team. These categories include: Number of subjects (scanned, excluded in database after acquisition), Inclusion criteria and descriptive statistics, Ethical considerations (for data collection centers), Design specifications, Task specification, Behavioral performance, All Acquisition Reporting, Preprocessing Reporting (for minimal processing pipeline), and Reproducibility (first level).

Finally, the following were not described in the present manuscript because they were not relevant to the present study: Statistical Modeling & Inference: Predictive analysis, and Results Reporting: Functional connectivity and Multivariate modelling & predictive analysis (these seem relevant but are not; see descriptions).

SUPPLEMENTAL RESULTS

1. Limitations in generalizability of ground truth and performance measures

Here, we used the full sample of the HCP dataset to generate a realistic definition of "ground truth" effects for benchmarking performance. The present designation of the full dataset as the "ground truth" population of interest implies that effects at all edges are considered to be exact, present, and nonzero (due to sampling variability) for the purpose of benchmarking. This is one of the largest task-based datasets collected for fMRI and provides one of the best empirical approximations of real effects to which we have access. However, this choice of ground truth limits the generalizability of the present results. We have previously noted a couple considerations about this measure of "ground truth" (21) and discuss those and additional caveats here.

The extent to which the benchmarking results generalize to the broader population beyond the sample used here is limited. Sign errors—differences between the estimated effect sign in the sample and the true effect sign in the population—are of particular concern since the ground truth effect sign is used for the definition of true and false positives. Sign errors are expected to be more likely for effects measured to be small in magnitude, and therefore measures of performance estimated for small effects in particular should be interpreted with caution. Given the large number of edges, it is likely that many estimated edge-level effects show particularly high errors in magnitude relative to the true population effect, so we may expect a number of sign errors for larger magnitude effects as well. The concern about sign errors is somewhat mitigated by the fact that the majority of edges and networks in the full sample were found to be significant (**Fig. 3**), lending more cautious confidence in the sign of these effects and their subsequent use in estimating performance.

Additionally, there is prominent family structure in the HCP associated with heritable task-based activation profiles (31). We did not use this nested structure in the estimation of effects and instead opted for a simple approach that treats all individuals as independent observations from a population. We anticipate that the unaccounted for dependence between individuals may bias the present estimates of ground truth effect sizes relative to the real task-based effects in the population. Furthermore, the fact that this dependence structure is not matched during resampling may further bias estimates of power. While we expect accounting for family structure to change estimates somewhat, we do not anticipate that it will substantially alter the main findings.

Finally, as always, the extent to which results generalize to other datasets, task contexts, study designs, processing strategies, etc. remains to be determined. We have elaborated on our expectations regarding the generalizability of these results in the **Discussion**. Notably, even though

we found similar benchmarking results for a sparser ground truth map (**SI Fig. 10**), there may be cases where effects are more focal than those found here that would benefit from more focal inferential strategies. We also note that while we employed regression of motion artifact and observe similar results whether motion is greater than, less than, or similar to task compared with rest (**SI Fig. 12**), there is always the potential for differences in motion or other artifacts to influence the observed ground truth effects, even with the best of mitigation strategies. Altogether, while we expect results to generalize to reasonably similar study designs, the spatial extent of effects and optimal level of inference for study designs that differ substantially is an open question.

2. Generalizability of the Shen268 partition to the HCP data

The Shen268 10 community atlas defined in the Yale High-Resolution Controls dataset was used to define a partition specifying 55 unique networks (see details in **SI Methods: 1.5**). We estimated whether this definition of networks showed substantially more between-network variability in the present data (HCP) than randomly defined networks. 10,000 permutations were used to test whether there was significant variability amongst 1) all 55 networks compared with randomly reassigning nodes across the 10 communities in the same partition (nodes rather than edges are shuffled in order to preserve the graph structure), 2) the 10 within-community networks compared with randomly shuffling edges across all within-community networks in the same partition, and 3) the 45 between-community networks compared with randomly shuffling edges across all between-community networks in the same partition. Greater variability was observed in all three cases for the original networks ($p<1E-10$; **Fig. 3d**). These findings are consistent with the idea that the original partition captures some of the structure in the present dataset, and not just because it separates within- and between-community networks.

We also estimated generalizability of the Shen268 partition to a partition derived from the HCP data using the Louvain method for community detection (community_louvain function in the Brain Connectivity Toolbox; (32); https://sites.google.com/site/bctnet/measures/list#TOC-Clustering-and-Community-Structure), which essentially forms communities by maximizing the sum of within-community weights. However, since task-rest contrasts generally showed negative edge weights within the Shen268 communities and positive weights between those communities, rest-task contrasts were instead used to facilitate comparison. Note that these edge weights represent *increased* connectivity during rest, rather than more direct measure of association typically used for community detection. Two sets of communities were estimated using two parameters specifying smaller ($\gamma=3$) and larger ($\gamma=4$) communities, and the “negative_asym” flag was provided to use

separate scalings for defining the modularity matrix using positive and negative edge weights. To obtain a stable definition of each set of communities, 10,000 iterations of this procedure were run and the most common assignment for each node was used as the final HCP-Louvain community assignment.

We then compared the overlap between the two sets of communities by 1) calculating which HCP-Louvain community that had the most nodes within each Shen268 community, then 2) calculating the proportion of nodes in that HCP-Louvain community that overlapped with the Shen268 community (**SI Fig. 4**). For the most part there appeared to be fair overlap between the sets of communities. A couple HCP-Louvain communities showed high overlap with a Shen268 community (Motor, V1, cerebellum). There were a few cases that suggested merging multiple of the Shen268 communities (e.g., merging frontoparietal, default mode, and VII), and several cases where there was relatively little unique overlap (e.g., VII and VAs, both some of the smallest communities). Altogether, despite being defined in a different dataset using rest-only data, the Shen268 partition seems to capture some of the structure of the task-related effects in the HCP and network-level pooling may be a meaningful place to start.

3. Differences between task and rest, and effect of unbalanced scan durations

All task and rest scans showed a similar spatial pattern of connectivity (**Fig. 3; SI Fig. 5a**). Connectivity was generally strong and positive within communities and often weaker or negative between communities. Effects associated with visual communities were particularly strong, with more positive connectivity amongst the visual communities and more negative connectivity between visual and other cortical communities. In contrast, subcortical and cerebellar connectivity was generally relatively weak.

The full resting scan showed stronger connectivity overall than any of the tasks, especially within-community (**SI Fig. 5**). However, rest was the longest of the scans (1200 frames), about triple that of the shortest task (176 frames in the EMOTION task). Since scan duration is directly related to the power to estimate connectivity (see also (33)), we estimated how effect sizes would change with rest trimmed to a duration matching the shortest task (176 frames; **SI Fig. 5**; detailed contrasts in **SI Fig. 6**). Resting state scans at either duration generally showed more positive connectivity within community and between motor-visual communities than task. Yet the trimmed rest scan showed weaker connectivity than untrimmed rest, and the pattern of increased connectivity between communities during rest compared with task became less clear with trimmed rest. While we don't expect this to substantially alter the main findings and rest remains consistently distinct from task

even at this shortest of scan durations (compare task-task with task-trimmed rest contrasts in **SI Fig. 6b**), care should be taken to account for differences in the scan duration that can bias contrasts towards the longer scan.

4. Nonparametric edge-level FDR correction implemented in the *NBS* toolbox

As indicated in the main text, all approaches were expected to control FWER in the weak sense, defined as attaining FWER levels below the upper bound of expected FWER when the null is true everywhere (95% confidence interval for FWER=3-7% for 500 repetitions; (34, 35)). This includes FDR controlling procedures (4). Unfortunately, the nonparametric edge-level FDR controlling procedure implemented in the *NBS* toolbox is expected to require many more permutations than is feasible for the present study to achieve valid control—namely, K=10^6–10^7 permutations (https://www.nitrc.org/forum/message.php?msg_id=31971; https://www.nitrc.org/forum/forum.php?thread_id=4543&forum_id=3444. This procedure estimates the null for each edge via permutation, then uses the Simes algorithm (12) for FDR correction. The time is takes to perform a single repetition of the present benchmarking experiment increases substantially with the number of permutations, with K=10^3 requiring 2 hours per repetition and a K=5x10^4 requiring 2 days per repetition. FWER decreased accordingly as expected—from FWER=100% (K=10^3) to FWER=20% (K=5x10^4)—so we expect that valid FWER can be achieved with K=10^6–10^7 permutations. However, this is not feasible in the current setting where each inferential procedure is evaluated with 24 experiments (8 tasks and 3 group sizes) and 500 repetitions per experiment. As a historical note, this inferential procedure was originally designed for use with relatively small networks which required fewer permutations and fewer resources per permutation.

One option for users to avoid invalid FWER control is to set the minimum estimable p-value to 1/K rather than 0 (i.e., by changing line 89 on NBSfdr.m from "pvals=zeros(1,J)" to "pvals=ones(1,J)"). However, this impacts power and it is instead recommended to use sufficient permutations when using the above procedure.

SUPPLEMENTAL FIGURES

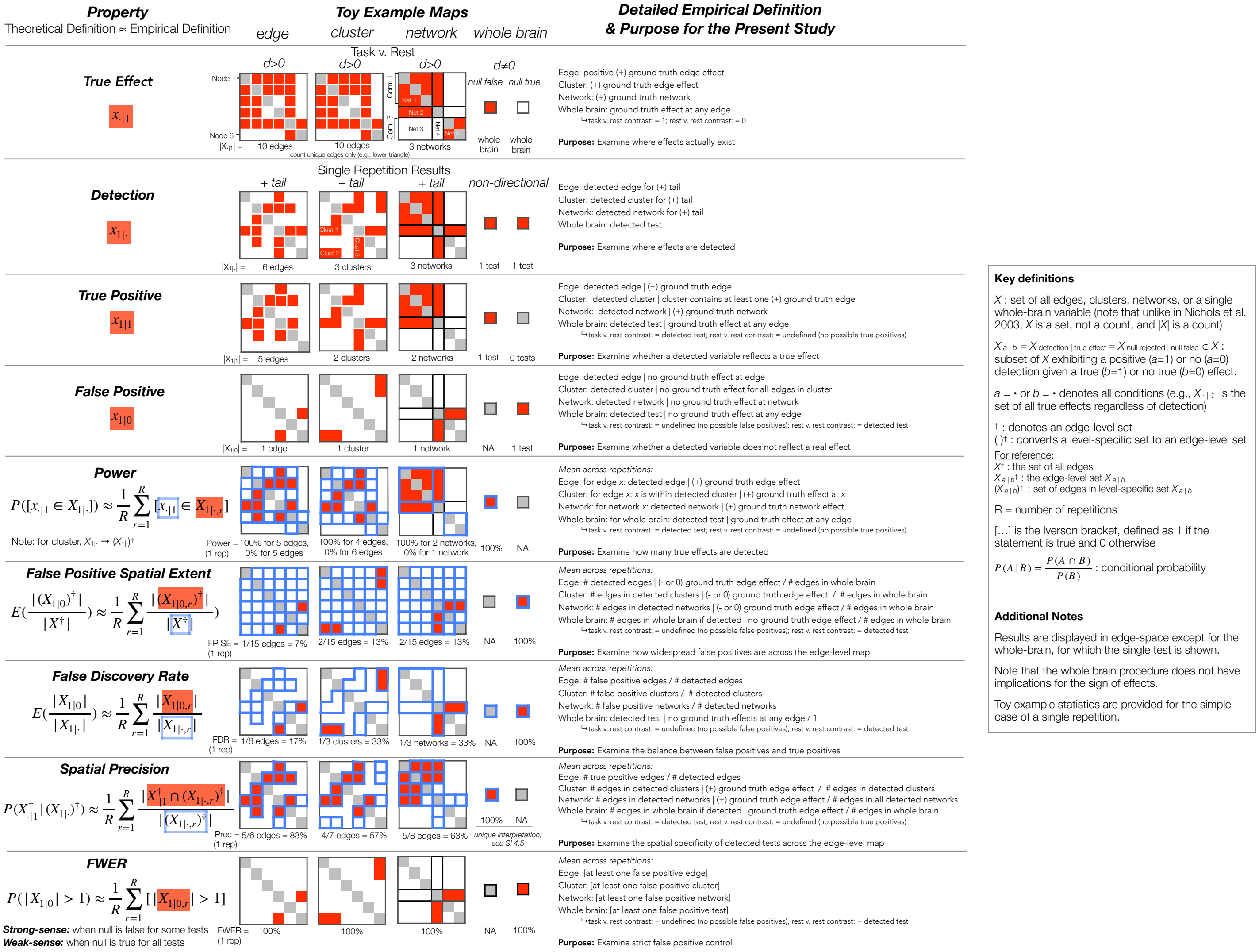

Supplemental Fig. 1. Error rate calculation. Left, theoretical definition and empirical definition (when appropriate) of each property. Middle, toy example maps illustrating components of the graph used to calculate respective error rates for edge, cluster, network, and whole-brain levels of inference. Areas highlighted in red and blue represent the subset and superset, respectively. The toy examples show results only for a single toy repetition and counts only include unique edges (e.g., for edge-level true effects, 10, not 20, edges are counted by counting the lower triangle only). Right, detailed empirical definition and purpose for the present study. Legend shows definitions of key terms and additional notes.

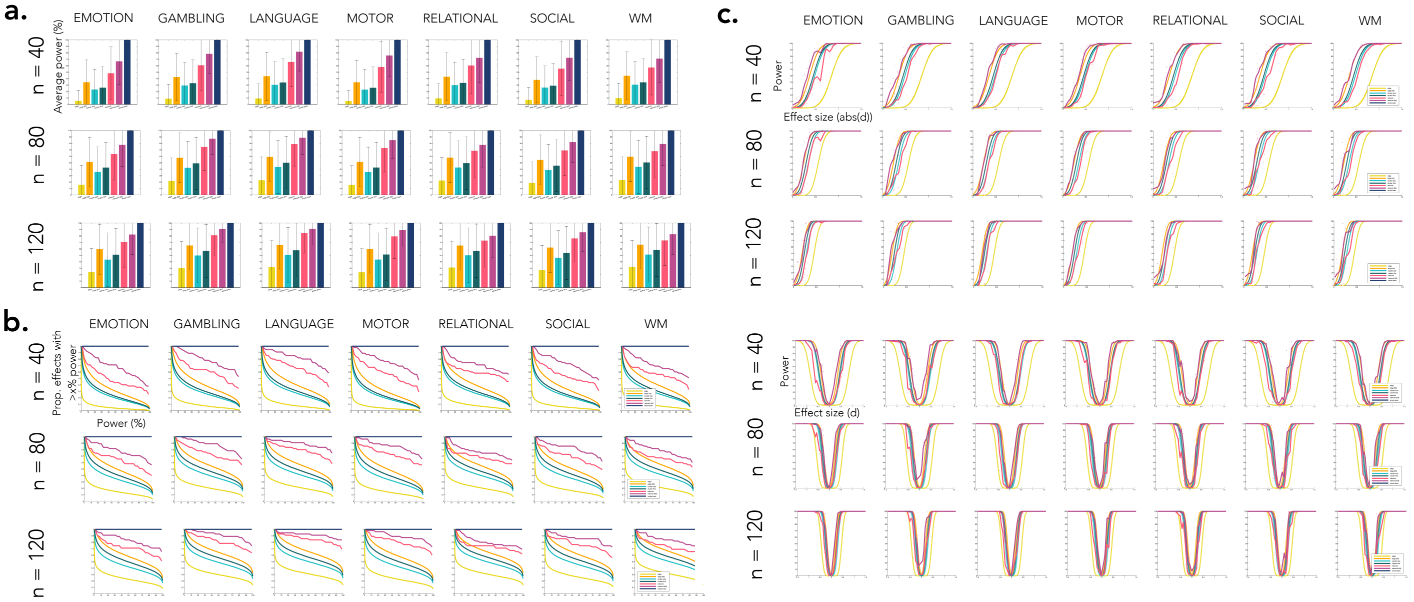

Supplemental Fig. 2. Power across levels of inference for individual tasks. Results from the following measures are shown at three sample sizes and for each of the seven tasks. The commonly targeted β=80% power threshold is indicated by the dashed grey line. **(a)** Average power to detect an effect (e.g., for "edge" procedure, mean power across all edges). Lines depict average across all tasks and bars depict standard error of the mean across tasks. **(b)** Proportion of effects exceeding each power level across all tasks. **(c)** Relationship between power and effect size for absolute value of effect size (top) and signed effect size (bottom).

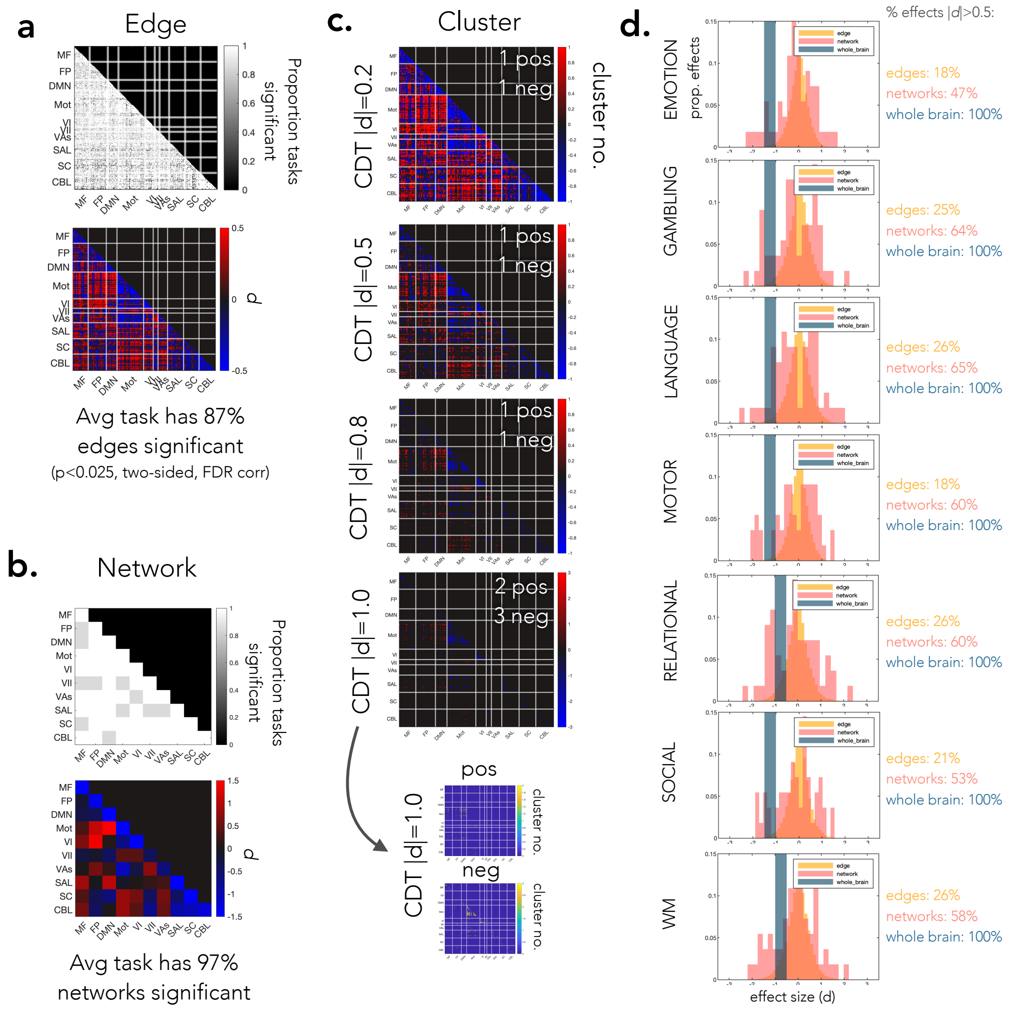

Supplemental Fig. 3. **Details of spatial extent of effects in the full "ground truth" dataset. (a)** Edge-level effect sizes and proportion of edges marked significant across tasks (p<0.05, two-sided t-test, FDR corrected). **(b)** Network-level effect sizes and proportion of networks marked significant across tasks (p<0.05, two-sided t-test, FDR corrected). Within-community connectivity, generally lower during task than rest, is highlighted by the yellow dotted rectangle. **(c)** Clusters extents across cluster determining thresholds. All edges surviving a cluster-determining threshold of |d|=0.2 (small; i.e., edges with *d*>0.2 and *d*<-0.2), 0.5 (medium), 0.8 (large), and 1.0 (very large) are shown and clusters of contiguous edges are counted. Only a single very large cluster determining threshold (|d|=1.0) yields more than one cluster. The bottom pair of figures shows the two positive and three negative clusters reported at that threshold, which are mainly bound by the predefined network definitions. **(d)** Histograms of effect size at the edge-level (40 bins), pooled within networks (20 bins), and pooled across the whole brain (2 bins).

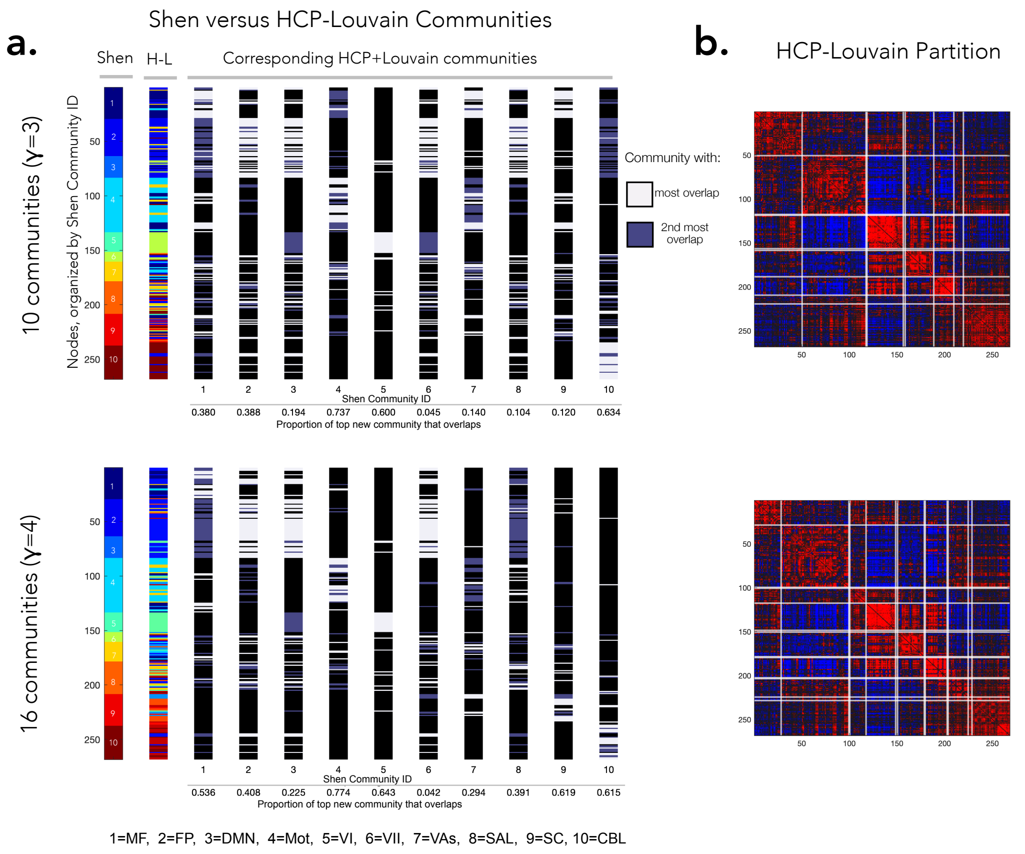

Supplemental Fig. 4. Generalizability of the Shen communities to communities estimated in the HCP data via the Louvain algorithm. Two parameters were used to estimate communities from the rest-mean task contrast: γ=3 (top; larger communities) and γ=4 (bottom; smaller communities). **(a)** For each bar, nodes are presented in the same order, grouped by the Shen 10 communities. The Shen communities are shown first, followed by the HCP Louvain communities. Subsequent bars to the left show the HCP-Louvain community which best overlaps with each Shen communities, defined as the community with the most nodes within the respective community. For example, the bar for Shen community #1 shows in white the nodes of the HCP-Louvain community that most overlap with Shen community #1. The second most overlapping community is also provided in dark grey. **(b)** HCP-Louvain graph partition based on reordering nodes into the newly estimated communities.

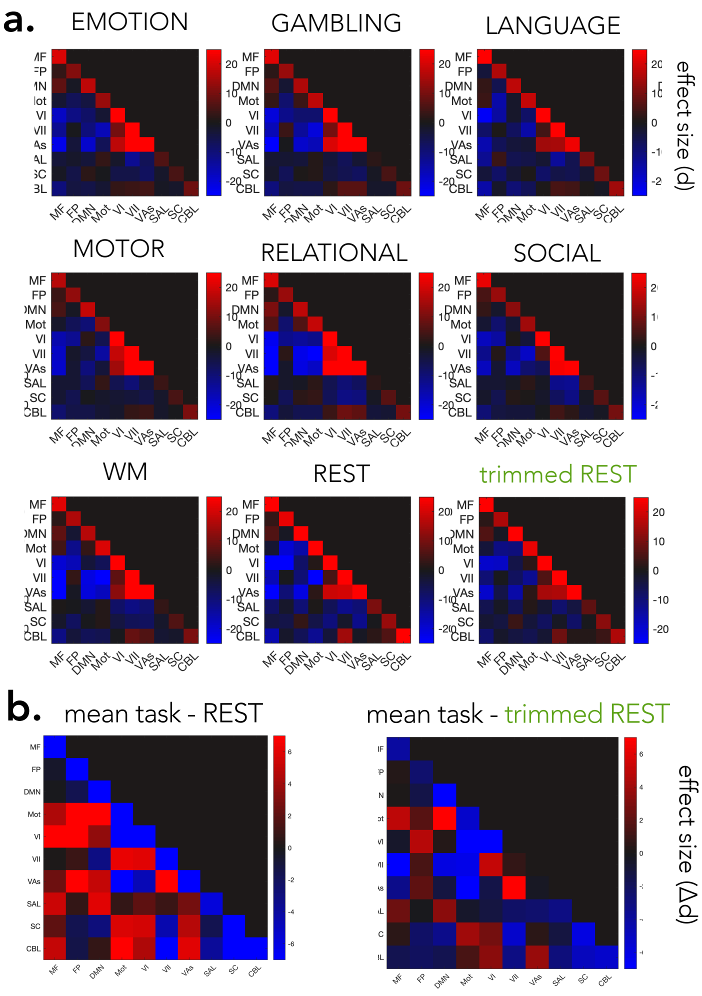

Supplemental Fig. 5. Comparison between all tasks and rest, both original and trimmed. (a) Effect size of each scan. **(b)** Difference between mean effect size across all tasks and rest effect size. Note that Δd = mean(task) d - rest d shown here is not the same as the mean d of the paired contrast between task and rest in the main text. For all plots, effects are calculated first at the edge level then averaged within networks. For trimmed rest results, rest was trimmed to match the shortest task (176 frames).

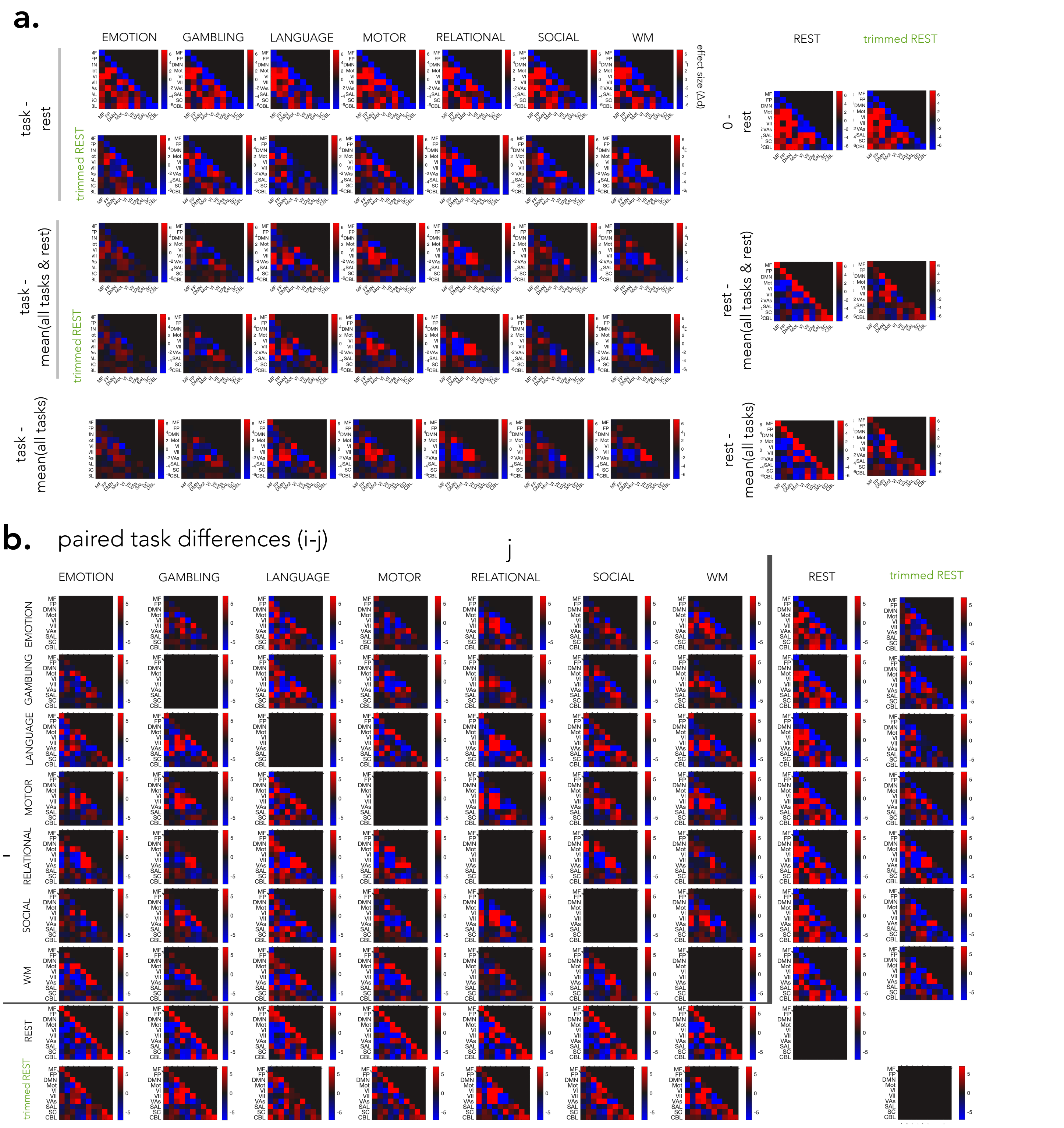

Supplemental Fig. 6. Detailed comparison between all tasks and rest, both original and trimmed. (a) Difference between each scan condition (task and rest) and various reference scans. Top, task minus rest. Middle, task minus mean of all tasks and rest. Bottom, task minus mean of all tasks, not including rest. Rows using trimmed rest noted in green. The right two columns show the difference between rest and various references. The same reference scans are used as above, except for the top row, which shows 0 minus rest. **(b)** Differences between all paired scan conditions. Differences are i - j, with i on the vertical axis and j on the horizontal axis. All effects are calculated first at the edge level then averaged within networks. For trimmed rest, rest was trimmed to match the shortest task (176 frames).

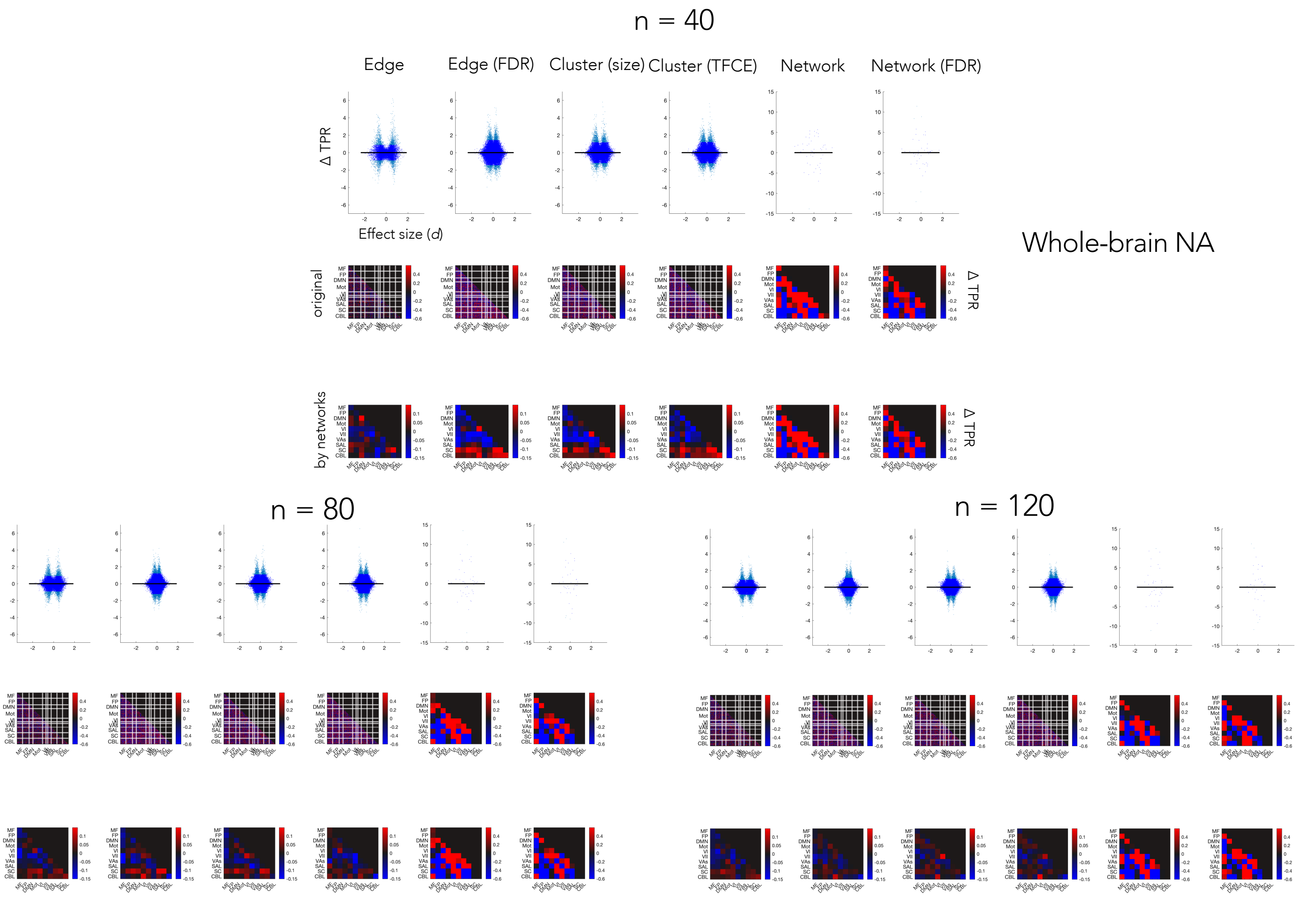

Supplemental Fig. 7. Spatial bias in power. Spatial and scatter plots show average task residuals of the effect size versus power curve. For each group size, the top row shows the relationship between residuals and effect size, the middle row shows the effects at the respective level of inference for each inferential procedure, and the bottom row shows the same results averaged within network. The distribution of results is not shown for the full brain procedure since there is only one data point (100% power for each group size).

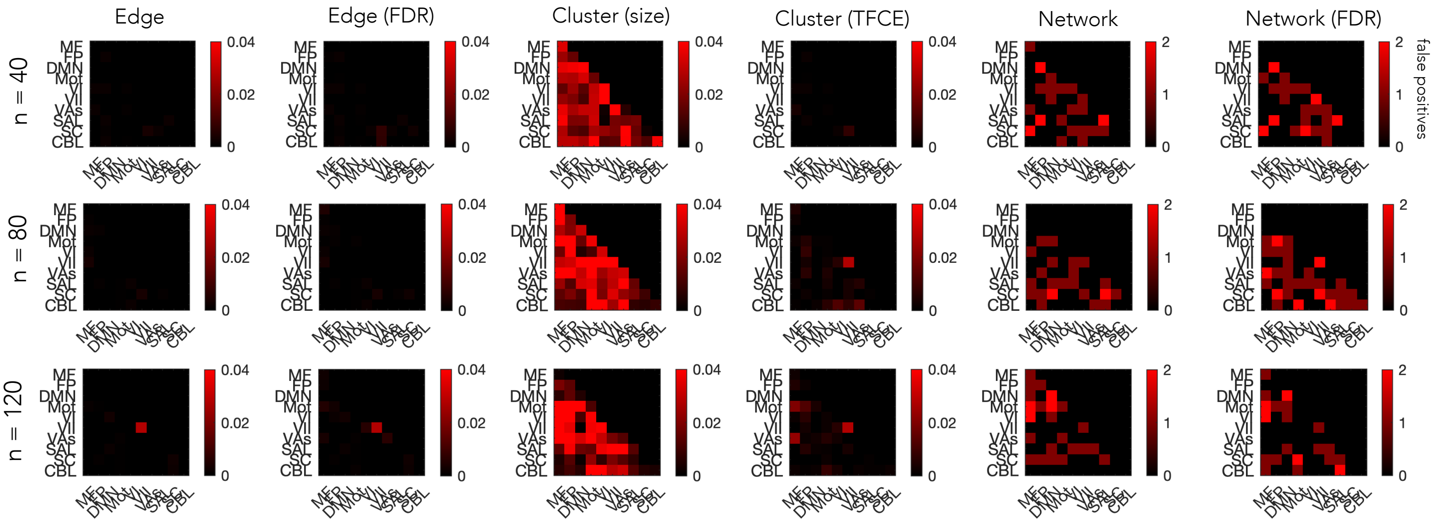

Supplemental Fig. 8. **Spatial bias in false positives.** Number of false positives for each inferential procedure, averaged within each network. Rows show results for each group size. The distribution of results is not shown for the full brain procedure since there is only one data point (0 false positives for each group size).

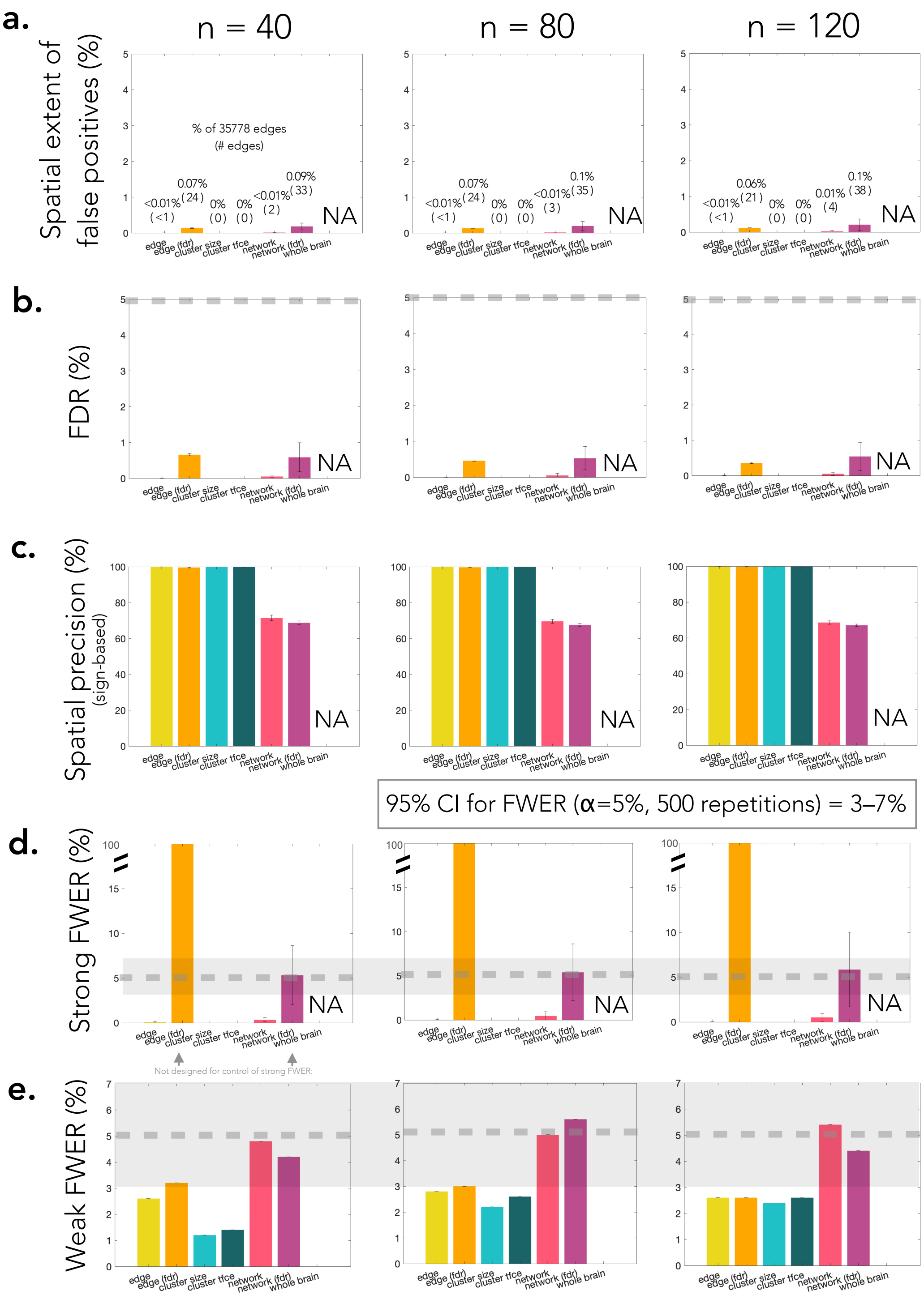

Supplemental Fig. 9. **Specificity across levels of inference for each sample size.** Results from the following measures at sample sizes n=40, n=80, and n=120, averaged across all seven tasks except as noted. **(a)** Spatial extent of false positives, defined as the proportion of the connectome showing false positives. **(b)** FDR, defined as the proportion of detections that are true positives. **(c)** Spatial precision, defined as the proportion of detections that overlap with "ground truth" effects in the same direction. FWER, strong-sense **(d)** and weak-sense (**e**; obtained using the "fake" task contrast), defined as the percent of repetitions with at least one false positive. The expected 95% CI for FWER is highlighted in grey, and valid control is defined as falling below the upper bound.

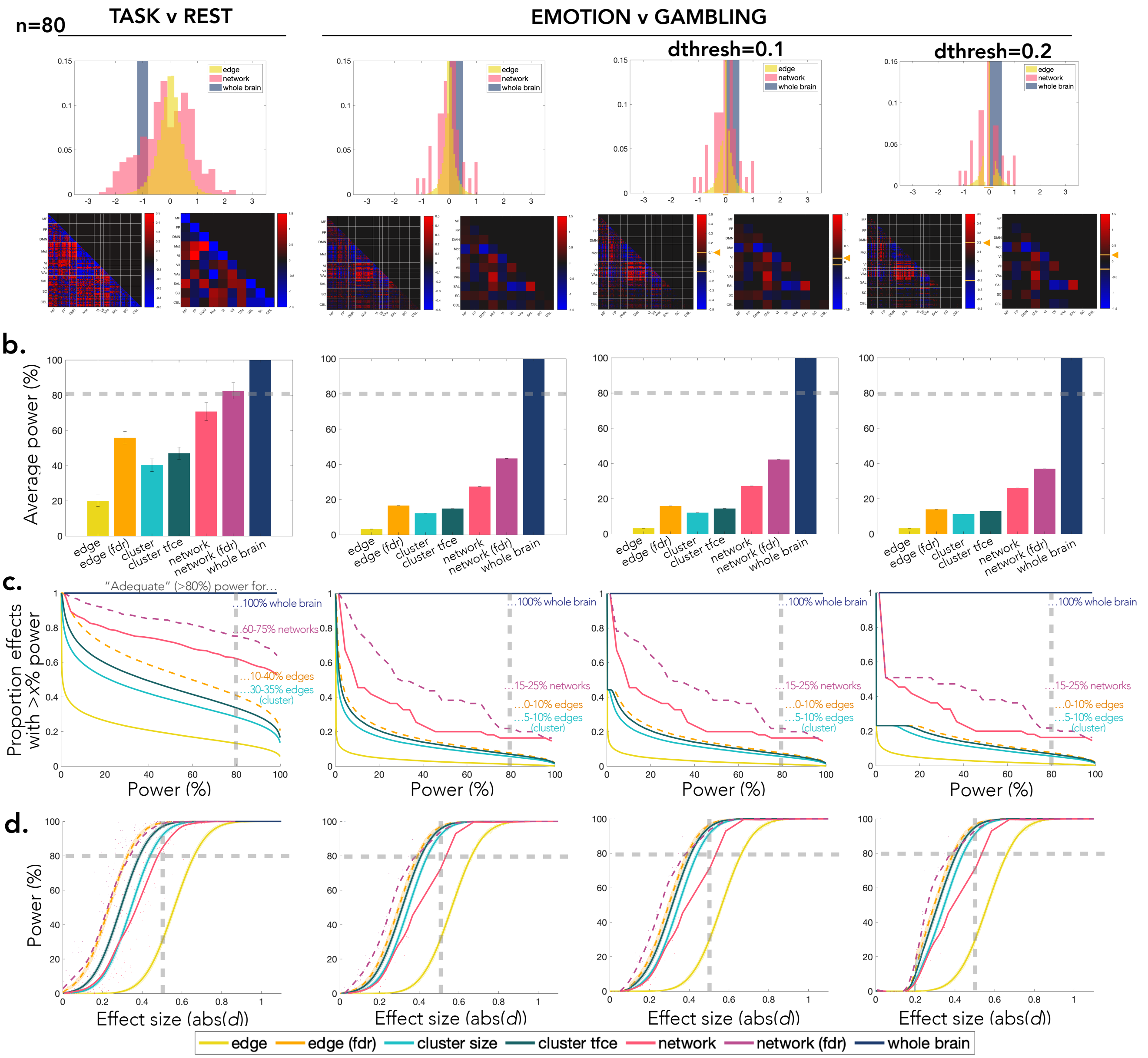

Supplemental Fig. 10. **Replication of sensitivity benchmarking results with weaker and sparser ground truth effects.** Effect size and sensitivity results for the Emotion versus Gambling contrast at three effect size thresholds to induce sparsity (d-threshold=[0,0.1,0.2]). Results from all task-versus-rest contrasts provided on the left for comparison. All results are shown for the n=80 sample size. The commonly targeted β=80% power threshold is indicated by the dashed grey line. **(a)** Effect size summaries. Histograms depict of effect size at the edge-level (40 bins), pooled within networks (20 bins), and pooled across the whole brain (2 bins). Matrices depict edge- (left) and network- (right) level effects. **(b)** Average power to detect an effect (e.g., for "edge" procedure, mean power across all edges). Bar heights depict average across all tasks and error bars depict standard error of the mean across tasks. **(c)** Proportion of effects exceeding each power level across all tasks. **(d)** Relationship between power and effect size for absolute value of effect size.

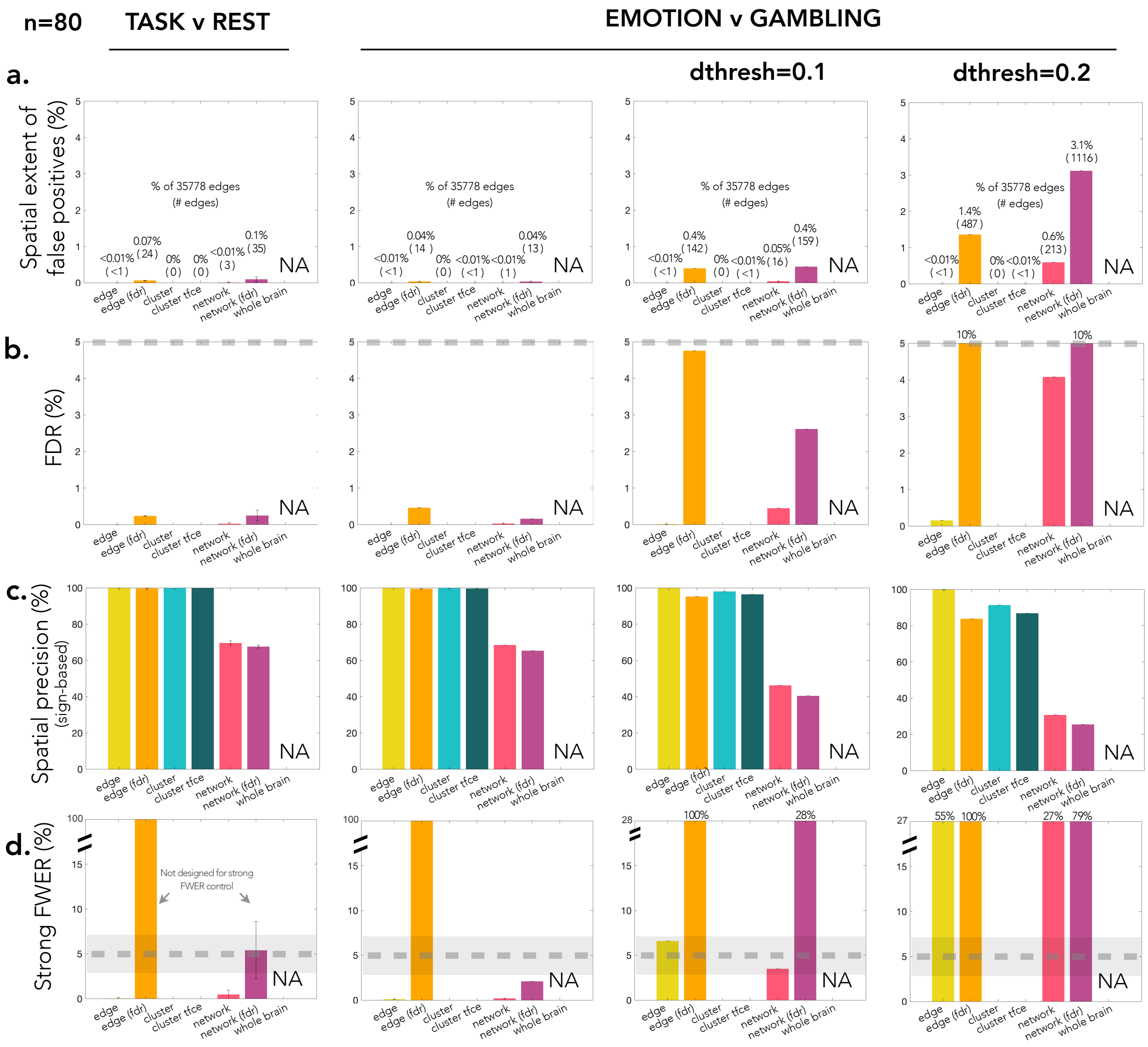

Supplemental Fig. 11. Replication of specificity benchmarking results with weaker and sparser ground truth effects. Specificity results for the Emotion versus Gambling contrast at three effect size thresholds to induce sparsity (d-threshold=[0,0.1,0.2]). Results from all task-versus-rest contrasts provided on the left for comparison. **(a)** Spatial extent of false positives, defined as the proportion of the connectome showing false positives. **(b)** FDR, defined as the proportion of detections that are true positives. **(c)** Spatial precision, defined as the proportion of detections that overlap with “ground truth” effects in the same direction. **(d)** Strong-sense FWER, defined as the percent of repetitions with at least one false positive. The expected 95% CI for FWER is highlighted in grey, and valid control is defined as falling below the upper bound.

Task vs. Rest1

Task	Motion Summary: Task			Motion Summary: Rest1			Task vs. Rest1 Contrast (t-test)			
	Mean	*Median*	*SD*	*Mean*	*Median*	*SD*	*P-value*	*T-statistic*	*95% CI (lower)*	*95% CI (upper)*
Emotion	0.0869	0.0788	0.0337	0.0864	0.0864	0.0339	0.5660	0.5742	-0.0011	0.0020
Gambling	0.0866	0.0779	0.0335	0.0869	0.0869	0.0347	0.6828	-0.4087	-0.0016	0.0010
Language	0.0899	0.0824	0.0333	0.0862	0.0862	0.0338	**8.7E-07**	4.9498	0.0022	0.0051
Motor	0.0953	0.0856	0.0367	0.0871	0.0871	0.0349	**2.7E-24**	10.4231	0.0066	0.0097
Relational	0.0916	0.0827	0.0390	0.0865	0.0865	0.0340	**2.1E-07**	5.2284	0.0032	0.0070
Social	0.0883	0.0805	0.0346	0.0862	0.0862	0.0337	**0.0065**	2.7272	0.0006	0.0036
Working Memory	0.0888	0.0807	0.0347	0.0872	0.0872	0.0349	**0.0193**	2.3442	0.0003	0.0029

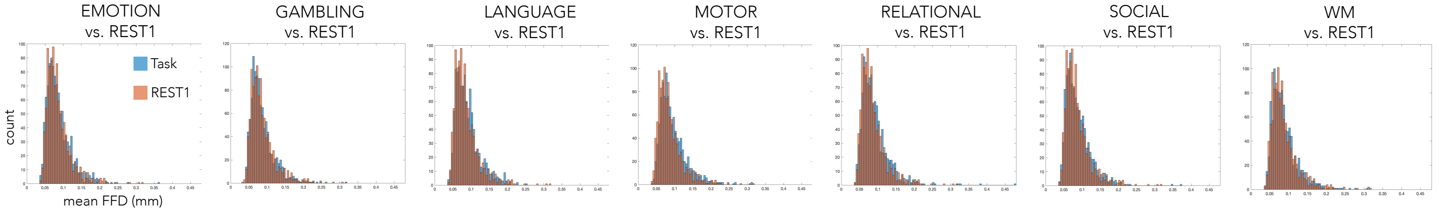

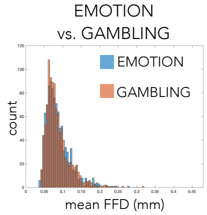

Emotion vs. Gambling

Motion Summary: Emotion			Motion Summary: Gambling			Emotion vs. Gambling Contrast (t-test)			
0.0865	0.0786	0.0326	0.0860	0.0860	0.0329	0.4699	0.7229	-0.0008	0.0018

Supplemental Fig. 12. Differences in motion between tasks used in contrasts. All results are based on mean frame-to-frame displacement (mm) for each subject (*Movement_RelativeRMS_mean.txt files from HCP). Top, Task versus Rest1 (corresponding with the main results); bottom, Emotion versus Gambling (corresponding with the associated supplemental result). The Motion Summary columns depict mean, median, and standard deviation of motion for each pair of tasks across all subjects who were included in the benchmarking analysis (note that subjects were only included in that analysis if they had all encoding scans for both that task and Rest1, yielding n>1000 subjects for each contrast). The Contrast columns depicts the results of a two-tailed t-test (p-value, t-statistic, and 95% confidence interval) comparing motion between each pair of tasks. Significant contrasts, uncorrected ($p<0.05$), are shown in bold. Histograms depict the distributions of subject motion for each pair of tasks in blue (main task) and red (reference task).

input : $\mathbf{Y} \in \mathbb{R}^{N\times E}$: N observations for E edges in a simple undirected graph;
$\mathbf{X} \in \mathbb{R}^{N\times M}$: design matrix for M parameters of interest;
$\mathbf{Z} \in \mathbb{R}^{N\times W}$: design matrix for W nuisance regressors (optional);
$\mathbf{c} \in \mathbb{R}^{M}$: contrast vector;
$\alpha \in \mathbb{R}$: target familywise error rate (FWER) threshold;
$K \in \mathbb{Z}$: number of permutations for FWER estimation;
$f_s : \mathbb{R}^E \longmapsto \mathbb{R}^E$: NBS estimator (see NBS methods I-III in section 2).
output : $\mathbf{s} \in \mathbb{R}^C$: NBS (stdNBS, tfNBS, or cNBS) for C components;
$\hat{\mathbf{h}}_1 \in \mathbb{R}^C$: positives (rejection of the null hypothesis after correction).
init : $\mathbf{P} \in \mathbb{R}^{N\times N\times K}$: {0,1} permutation matrix, where each $\mathbf{P}_k$ is a $N \times N$ matrix with a single 1 per row and column.

for $e \leq E$; **do** `// fit unpermuted model`

$\{\hat{\boldsymbol{\beta}}_e, \hat{\gamma}_e, \hat{\boldsymbol{\epsilon}}_e\} = \underset{\boldsymbol{\beta},\boldsymbol{\gamma}}{\operatorname{argmin}} ||\boldsymbol{\epsilon} = \mathbf{Y}_e - \mathbf{X}\boldsymbol{\beta} - \mathbf{Z}\boldsymbol{\gamma}||^2$; `// Least Squares`

$\{\hat{\gamma}_{\mathbf{Z},e}, \hat{\boldsymbol{\epsilon}}_{\mathbf{Z},e}\} = \underset{\gamma_{\mathbf{Z}}}{\operatorname{argmin}} ||\boldsymbol{\epsilon} = \mathbf{Y}_e - \mathbf{Z}\boldsymbol{\gamma}||^2$;

$\mathbf{t}_e \in \mathbb{R} = \dfrac{\mathbf{c}^{\intercal}\hat{\boldsymbol{\beta}}_e}{\sqrt{\hat{\boldsymbol{\epsilon}}_e^{\intercal}\hat{\boldsymbol{\epsilon}}_e \mathbf{c}^{\intercal}(\mathbf{X}^{\intercal}\mathbf{X})^{-1}\mathbf{c}/(N-M)}}$; `// t-statistic for contrast`

end

$\mathbf{s} = f_s(\mathbf{t})$;

$\mathbf{s}_{max} \in \mathbb{R} = \max(\mathbf{s})$;

for $p \leq K$; **do** `// fit permuted models`

for $e \leq E$; **do**

$\mathbf{Y}_e^* = \mathbf{P}_p \hat{\boldsymbol{\epsilon}}_{\mathbf{Z},e} + \mathbf{Z}\hat{\gamma}_{\mathbf{Z},e}$;

$\{\hat{\boldsymbol{\beta}}_e^*, \hat{\gamma}_e^*, \hat{\boldsymbol{\epsilon}}_e^*\} = \underset{\boldsymbol{\beta},\boldsymbol{\gamma}}{\operatorname{argmin}} ||\boldsymbol{\epsilon} = \mathbf{Y}_e^* - \mathbf{X}\boldsymbol{\beta} - \mathbf{Z}\boldsymbol{\gamma}||^2$;

$\mathbf{t}_e^* = \dfrac{\mathbf{c}^{\intercal}\hat{\boldsymbol{\beta}}_e^*}{\sqrt{\hat{\boldsymbol{\epsilon}}_e^{*\intercal}\hat{\boldsymbol{\epsilon}}_e^* \mathbf{c}^{\intercal}(\mathbf{X}^{\intercal}\mathbf{X})^{-1}\mathbf{c}/(N-M)}}$;

end

$\mathbf{s}_p^* = f_s(\mathbf{t}^*)$;

$\mathbf{s}_{max,p}^* \in \mathbb{R} = \max(\mathbf{s}_p^*)$;

end

if f_s is cNBS **then**

$\mathbf{p}_c = \dfrac{\sum_{k=1}^{K} \mathbf{s}_c \leq \mathbf{s}_{c,k}^*}{K}$; `// uncorrected p-value`

$\mathbf{p}_c' = \mathbf{p}_c * C$; `// FWER-corrected p-value (Bonferroni)`

else

$\mathbf{p}_c' = \dfrac{\sum_{k=1}^{K} \mathbf{s}_c \leq \mathbf{s}_{max,k}^*}{K}$; `// FWER-corrected p-value`

end

$\hat{\mathbf{h}}_{1,c} = [\mathbf{p}_c' \leq \alpha]$. `// where [...] is the Iverson bracket`

Supplemental Fig. 13. NBS, tfNBS, and cNBS algorithm reprinted from Noble et al., 2020, in MICCAI with permission. NBS is analogous to the “cluster” procedure, tfNBS is analogous to the “cluster TFCE” procedure, and cNBS is analogous to the “network” procedure. **s** is the network-based statistic specific to each procedure; for cNBS, $\mathbf{s}_{\mathrm{c}} = \frac{\sum_{\mathrm{e}=1}^{\mathrm{E}'} \mathbf{t}'_{\mathrm{c,e}}}{|\mathbf{E}'_{\mathrm{c}}|}$, where **t** represents the weights of graph **U**(**V**,**E**,**t**) (all graphs **U** represent simple undirected graphs of **V** nodes and **E** edges without loops) and **U**’ represents a partition of **U**. The “network (FDR)” procedure follows the same algorithm except the correction procedure (line 19) uses the Storey algorithm to control FDR. The “whole brain” procedure also follows the same algorithm except that $\mathbf{p} = \frac{\sum_{k=1}^{K} \mathbf{s} \leq \mathbf{s}*_k}{K}$ (line 18) and only a single test is used so no multiple comparison correction is applied (line 19). cNBS has been introduced in Noble et al., 2020, and mv-cNBS is introduced here; the other procedures have been described in detail elsewhere (see **SI Methods**).

SUPPLEMENTAL REFERENCES

1. A. Zalesky, A. Fornito, E. T. Bullmore, Network-based statistic: identifying differences in brain networks. *Neuroimage* **53**, 1197-1207 (2010).
2. J. M. Bland, D. G. Altman, Multiple significance tests: the Bonferroni method. *BMJ* **310**, 170 (1995).
3. J. D. Storey, A direct approach to false discovery rates. *Journal of the Royal Statistical Society: Series B (Statistical Methodology)* **64**, 479-498 (2002).
4. Y. Benjamini, Y. J. J. o. t. R. s. s. s. B. Hochberg, Controlling the false discovery rate: a practical and powerful approach to multiple testing. **57**, 289-300 (1995).
5. S. M. Smith, T. E. Nichols, Threshold-free cluster enhancement: addressing problems of smoothing, threshold dependence and localisation in cluster inference. *Neuroimage* **44**, 83-98 (2009).
6. H. C. Baggio *et al.*, Statistical inference in brain graphs using threshold-free network-based statistics. **39**, 2289-2302 (2018).
7. T. Spisak *et al.*, Probabilistic TFCE: A generalized combination of cluster size and voxel intensity to increase statistical power. *Neuroimage* **185**, 12-26 (2019).
8. J.-D. Tournier *et al.*, MRtrix3: A fast, flexible and open software framework for medical image processing and visualisation. *Neuroimage* **202**, 116137 (2019).
9. L. Vinokur, A. Zalesky, D. Raffelt, R. Smith, A. Connelly, A Novel Threshold-Free Network-Based Statistics Method: Demonstration using Simulated Pathology. *Organization for Human Brain Mapping*, 4144 (2015).
10. S. Noble, D. Scheinost, The constrained network-based statistic: a new level of inference for neuroimaging. *Medical Image Computing and Computer Assisted Intervention* (2020).
11. X. Shen, F. Tokoglu, X. Papademetris, R. T. Constable, Groupwise whole-brain parcellation from resting-state fMRI data for network node identification. *Neuroimage* **82**, 403-415 (2013).
12. R. J. Simes, An improved Bonferroni procedure for multiple tests of significance. *Biometrika* **73**, 751-754 (1986).
13. N. Pike, Using false discovery rates for multiple comparisons in ecology and evolution. *Methods in ecology and Evolution* **2**, 278-282 (2011).
14. D. C. Van Essen *et al.*, The WU-Minn Human Connectome Project: an overview. *Neuroimage* **80**, 62-79 (2013).
15. S. M. Smith *et al.*, Resting-state fMRI in the human connectome project. *Neuroimage* **80**, 144-168 (2013).
16. A. S. Greene, S. Gao, S. Noble, D. Scheinost, R. T. Constable, How tasks change whole-brain functional organization to reveal brain-phenotype relationships. *Cell reports* **32**, 108066 (2020).
17. M. F. Glasser *et al.*, The minimal preprocessing pipelines for the Human Connectome Project. *Neuroimage* **80**, 105-124 (2013).
18. D. M. Barch *et al.*, Function in the human connectome: task-fMRI and individual differences in behavior. *Neuroimage* **80**, 169-189 (2013).
19. A. Joshi *et al.*, Unified framework for development, deployment and robust testing of neuroimaging algorithms. *Neuroinformatics* **9**, 69-84 (2011).
20. T. D. Satterthwaite *et al.*, An improved framework for confound regression and filtering for control of motion artifact in the preprocessing of resting-state functional connectivity data. *Neuroimage* **64**, 240-256 (2013).
21. S. Noble, D. Scheinost, R. T. Constable, Cluster failure or power failure? Evaluating sensitivity in cluster-level inference. *Neuroimage* **209**, 116468 (2020).
22. H. R. Cremers, T. D. Wager, T. Yarkoni, The relation between statistical power and inference in fMRI. *PloS one* **12**, e0184923 (2017).
23. A. Eklund, T. E. Nichols, H. Knutsson, Cluster failure: why fMRI inferences for spatial extent have inflated false-positive rates. *Proceedings of the National Academy of Sciences*, 201602413 (2016).

24. A. S. Greene, S. Gao, S. Noble, D. Scheinost, R. T. Constable (2019) How Tasks Change Whole-Brain Functional Organization to Reveal Brain-Phenotype Relationships. in *NEURON-D-19-01606*.
25. J. Cohen, *Statistical power analysis for the behavioral sciences* (Academic press, 2013).
26. R. A. Poldrack *et al.*, Scanning the horizon: towards transparent and reproducible neuroimaging research. *Nat Rev Neurosci* **18**, 115-126 (2017).
27. D. Szucs, J. P. Ioannidis, Sample size evolution in neuroimaging research: An evaluation of highly-cited studies (1990–2012) and of latest practices (2017–2018) in high-impact journals. *NeuroImage* **221**, 117164 (2020).
28. T. Nichols, S. Hayasaka, Controlling the familywise error rate in functional neuroimaging: a comparative review. *Statistical methods in medical research* **12**, 419-446 (2003).
29. C.-W. Woo, A. Krishnan, T. D. Wager, Cluster-extent based thresholding in fMRI analyses: pitfalls and recommendations. *Neuroimage* **91**, 412-419 (2014).
30. K. J. Friston, A. Holmes, J. B. Poline, C. J. Price, C. D. Frith, Detecting activations in PET and fMRI: levels of inference and power. *Neuroimage* **4**, 223-235 (1996).
31. Y. Benhajali *et al.*, Subtypes of brain activation are heritable and genetically linked with behavior in the Human Connectome Project sample. (2020).
32. M. Rubinov, O. Sporns, Complex network measures of brain connectivity: uses and interpretations. *Neuroimage* **52**, 1059-1069 (2010).
33. J. W. Cho, A. Korchmaros, J. T. Vogelstein, M. Milham, T. Xu, Impact of Concatenating fMRI Data on Reliability for Functional Connectomics. *BioRxiv* (2020).
34. E. B. Wilson, Probable inference, the law of succession, and statistical inference. *Journal of the American Statistical Association* **22**, 209-212 (1927).
35. A. M. Winkler, G. R. Ridgway, M. A. Webster, S. M. Smith, T. E. Nichols, Permutation inference for the general linear model. *Neuroimage* **92**, 381-397 (2014).
